# A Multi-Scale Model of Hepcidin Promoter Regulation Reveals Factors Controlling Systemic Iron Homeostasis

**DOI:** 10.1371/journal.pcbi.1003421

**Published:** 2014-01-02

**Authors:** Guillem Casanovas, Anashua Banerji, Flavia d'Alessio, Martina U. Muckenthaler, Stefan Legewie

**Affiliations:** 1 Department of Pediatric Oncology, Hematology and Immunology, University Hospital of Heidelberg, Heidelberg, Germany; 2 Molecular Medicine Partnership Unit, Heidelberg, Germany; 3 European Molecular Biology Laboratory, Heidelberg, Germany; 4 Institute of Molecular Biology (IMB), Mainz, Germany; 5 BioQuant, Heidelberg, Germany; Brigham & Women's Hospital and Harvard Medical School, United States of America

## Abstract

Systemic iron homeostasis involves a negative feedback circuit in which the expression level of the peptide hormone hepcidin depends on and controls the iron blood levels. Hepcidin expression is regulated by the BMP6/SMAD and IL6/STAT signaling cascades. Deregulation of either pathway causes iron-related diseases such as hemochromatosis or anemia of inflammation. We quantitatively analyzed how BMP6 and IL6 control hepcidin expression. Transcription factor (TF) phosphorylation and reporter gene expression were measured under co-stimulation conditions, and the promoter was perturbed by mutagenesis. Using mathematical modeling, we systematically analyzed potential mechanisms of cooperative and competitive promoter regulation by the transcription factors, and experimentally validated the model predictions. Our results reveal that hepcidin cross-regulation primarily occurs by combinatorial transcription factor binding to the promoter, whereas signaling crosstalk is insignificant. We find that the presence of two BMP-responsive elements enhances the steepness of the promoter response towards the iron-sensing BMP signaling axis, which promotes iron homeostasis *in vivo*. IL6 co-stimulation reduces the promoter sensitivity towards the BMP signal, because the SMAD and STAT transcription factors compete for recruiting RNA polymerase to the transcription start site. This may explain why inflammatory signals disturb iron homeostasis in anemia of inflammation. Taken together, our results reveal why the iron homeostasis circuit is sensitive to perturbations implicated in disease.

## Author Summary

The nutritional iron uptake is tightly regulated because the body has limited capacity of iron excretion. Mammals maintain iron homeostasis by a negative feedback loop, in which the peptide hepcidin senses the iron blood level and controls iron resorption. Molecular perturbations in the homeostasis loop lead to iron-related diseases such as hemochromatosis or anemia of inflammation. Quantitative studies are required to understand the dynamics of the iron homeostasis circuitry in health and disease. We investigated how the biological activity of hepcidin is regulated by combining experiments with mathematical modeling. We present a multi-scale model that describes the signaling network and the gene promoter controlling hepcidin expression. Possible scenarios of hepcidin regulation were systematically tested against experimental data, and interpreted using a network model of iron metabolism *in vivo*. The analysis showed that the presence of multiple redundant regulatory elements in the hepcidin gene promoter facilitates homeostasis, because changes in iron blood levels are sensed with high sensitivity. We further suggest that inflammatory signals establish molecular competition at the hepcidin promoter, thereby reducing its iron sensitivity and leading to a loss of homeostasis in anemia of inflammation. We conclude that quantitative insights into hepcidin expression regulation explain features of systemic iron homeostasis.

## Introduction

Hepcidin is a humoral polypeptide that plays a central role in systemic iron homeostasis (reviewed in [1]). One main function of hepcidin is to maintain constant levels of iron circulating in the blood despite imbalances in external iron availability: Iron overload in the blood stimulates hepcidin transcription in hepatocytes. Hepcidin in turn blocks intestinal iron uptake and macrophage iron release into the blood by binding to the iron exporter ferroportin and triggering its degradation. Thus, hepcidin is part of a negative feedback circuit that stabilizes the iron blood concentration.

Negative feedback is known to play a key role for the robustness and homeostasis of biochemical networks (reviewed in [2]). Biochemical negative feedbacks have been shown to compensate for various perturbations including stochasticity in gene expression [3], [4], mutations [5] and pharmacological inhibition [6], [7].

In systemic iron homeostasis, iron diet content affects the iron concentration in the blood [8], [9], suggesting that the hepcidin feedback loop only partially compensates for perturbations. Nevertheless, genetic perturbations of iron-dependent hepcidin regulation result in hemochromatosis, a common hereditary disease [10]. At the molecular level, hereditary hemochromatosis (HH) is caused by inappropriately low hepcidin expression and an inability to compensate changes in iron blood levels. HH patients are characterized by chronic iron overload that causes organ damage such as liver fibrosis.

Hepcidin expression is primarily controlled at the transcriptional level. Information about iron blood levels is transduced from the hepatocyte cell membrane to the nucleus by the bone morphogenetic protein (BMP) signalling pathway [11], [12]: Increasing iron blood concentrations are sensed by Hfe and Tfr2, two transmembrane proteins mutated in HH [13], [14]. The signal is transmitted by the BMP co-receptor HJV and BMP receptor 1 to trigger the phosphorylation and nuclear translocation of SMAD1/5/8 transcription factors (referred to as SMADs hereafter). BMP6 is regulated by hepatic iron levels and plays a critical role in this process [15], [16]. The hepcidin promoter contains two BMP-responsive elements (BRE1 and BRE2) that are recognized by the SMADs (sometimes also abbreviated as BMP-RE1 and BMP-RE2) [17]–[19]. Mutations in the BRE1 promoter element and in the BMP signalling pathway are associated with HH [20].

Hepcidin expression is also regulated by inflammatory cytokines and hypoxia [21], [22]. Inflammatory cytokines such as IL6 activate the STAT3 signaling pathway in hepatocytes. Phosphorylated STAT3 transcription factors (TFs) are directly recruited to a STAT binding site (STATBS) in the hepcidin promoter, thereby enhancing hepcidin expression and reducing iron blood levels [23], [24]. Chronic inflammation causes an iron-related disorder, known as anemia of inflammation (AI), because the persistent lack of iron availability blocks erythropoiesis [10]. This indicates that the integration of BMP and IL6 signals at the level of hepcidin expression plays a key role in systemic iron homeostasis.

Combinatorial gene regulation by binding of multiple different transcription factors to the same promoter is a recurrent aspect of transcription networks. Thermodynamic modeling employs methods from statistical thermodynamics to describe combinatorial binding of transcription factors and RNA polymerase (RNAP) to the promoter, depending on the protein concentrations and binding energies. [25]–[32]. The modeling framework focusses on transcription initiation and is based on the assumption that gene activity is determined by RNAP recruitment to the transcription start site (TSS). Thermodynamic modeling has been shown to accommodate various modes of signal integration on a promoter [25], [26], [29], some of which have been confirmed experimentally for bacterial and yeast promoters [33]–[36]. More recently, the framework was extended to aspects of eukaryotic gene regulation, including nucleosome positioning effects [37], [38].

In this work, we combined experimental measurements and thermodynamic modeling to quantitatively analyze how the iron-sensing BMP and inflammatory IL6 pathways coordinately control hepcidin expression.

## Results

### BMP-responsive elements 1 and 2 play different roles in the regulation of hepcidin expression

Systemic iron homeostasis is maintained by an auto-regulatory negative feedback loop that involves the transcriptional induction of hepcidin in the liver: elevated iron levels in the blood induce hepcidin expression by activating the BMP signaling pathway in the liver. Released hepcidin in turn induces the degradation of intestinal iron transporters, thereby lowering the blood iron level. We analyzed a conceptual mathematical model of this circuitry to understand how systemic iron homeostasis can be maintained despite imbalances in iron availability and consumption (Supplemental Text S1). The model suggests that the regulatory loop most potently balances iron blood levels if hepcidin expression responds in a steep, nonlinear manner to alterations in iron blood levels. Robust homeostasis further requires that the hepcidin promoter is able to sense and to respond to a broad range of iron blood concentrations. We therefore reasoned that data of dose-dependent hepcidin promoter regulation, and its modulation by inflammatory IL6 stimulation, could provide valuable insights into the regulation of iron homeostasis.

**Figure 1:**
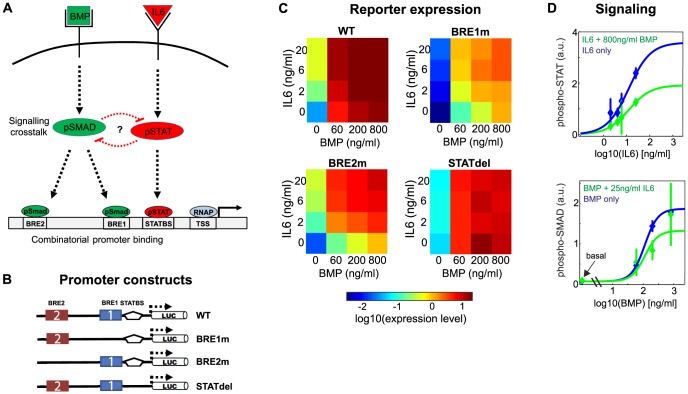
Signal integration at the hepcidin promoter. (**A**) Schematic representation of two critical signaling pathways controlling hepcidin expression. SMAD and STAT transcription factors are phosphorylated upon BMP and IL6 stimulation, and bind BMP-responsive elements (BRE) and a STAT-binding site (STATBS) in the hepcidin promoter, respectively. The importance of signaling crosstalk is not clear. (TSS: transcription start site; RNAP: RNA polymerase) (**B**) and (**C**) Analysis of transcription factor crosstalk at the promoter level by reporter gene assays. Luciferase expression is driven by the wildtype (WT) hepcidin promoter (3 kb upstream of TSS) or promoter mutants lacking one of the transcription factor binding sites (panel B; BRE1m = BRE1 mutated; STATdel = deleted for STATBS). Luciferase activity of each reporter construct (shown on a log10-scale) was measured for increasing doses of IL6 and/or BMP (n = 6). All heatmaps represent the mean of at least four biological replicates (see Methods), and are given in the same arbitrary concentration units (**D**) Moderate inhibitory signaling crosstalk at the signaling level. Immunoblots against phosphorylated SMAD1/5/8 and STAT3 (Supplemental Fig. S2) were quantified by densitometric analysis. The data points and error bars represent mean and standard deviation of biological replicates (N = 2), respectively (see Methods). Lines are fits of the sigmoidal Hill equation (y = y_basal_ + y_max_ * S^n^/(S^n^ + EC_50_^n^), S…stimulus, y_basal_…basal signaling activity, y_max_…maximal pathway activation, EC50….half-maximal-stimulus, n…Hill coefficient). The fits with and without non-canonical stimulation (blue and green lines, respectively) solely differ in the y_max_ values.

Hepatic cell culture systems do not directly respond to stimulation with extracellular iron [39]. BMP6 is involved in hepatic iron-sensing *in vivo*, and is commonly used as an external stimulus to characterize how hepcidin expression responds to changes in iron blood levels *in vitro* [15], [16]. We performed co-stimulation experiments with BMP6 and IL6 in human hepatoma (HuH7) cells, and measured the activity of a luciferase reporter gene driven by the hepcidin promoter 24 h after stimulation. The hepcidin expression response of HuH7 cells was characterized in previous studies, and it was concluded that HuH7 cells reflect known features of hepcidin expression *in vivo* (see Discussion). Reporter gene assays were performed in HuH7 cells transiently expressing luciferase constructs under control of wildtype (WT) or mutant hepcidin promoters (Fig. 1B and C). The WT promoter spans 3 kb upstream of the transcription start site, and contains a proximal STAT-binding site (STATBS), a nearby BMP-responsive element (BRE1), and a distal BMP-responsive element (BRE2). We and others previously showed that these sequence motifs are necessary and sufficient for responsiveness towards BMP and IL6 stimulation (see Discussion). In each of the three promoter mutants, one of these transcription factor binding sites is non-functional: The BRE1m and BRE2m constructs exhibit point mutations in the corresponding BMP-responsive elements, while STATdel is characterized by a complete deletion of the STAT-binding site. For simplicity, we will generally refer to transcription binding site mutations even when discussing the deletion of the STATBS.

The raw luciferase activity reads of at least four biological replicates were processed (see Methods) and the merged data is shown in Fig. 1C (using the same arbitrary concentration units for all heatmaps). BMP6 and IL6 mono-stimulation both increased the luciferase activities of the WT promoter construct. Maximal IL6 stimulation enhanced luciferase activities by 20-fold, while a 450-fold increase was observed upon maximal BMP stimulation. The IL6 response saturated within the concentration range used, because IL6 concentrations beyond 6 ng/ml hardly increased expression any further. Thus, BMP6 increased expression much more efficiently than IL6 in terms of maximal possible inducibility.

Co-stimulation with IL6 and BMP further enhanced the luciferase activity of the WT promoter compared to mono-stimulation. We confirmed by qPCR measurements that this co-stimulation response quantitatively reflects the expression of endogenous hepcidin mRNA (Supplemental Protocol S1, Supplemental Fig. S1).

As expected, the STATdel promoter fails to respond to IL6 stimulation, but is still sensitive to BMP treatment. The luciferase activities of the BRE1m construct are much lower than WT for both basal and induced conditions (Fig. 1C). However, the BRE1m promoter is qualitatively similar to the WT promoter in terms of stimulus inducibility, because maximal BMP stimulation induces a large increase in luciferase activity, while the maximal IL6 dose has a much lesser impact.

The co-stimulation response of the BRE2m construct is qualitatively different from that of the WT and BRE1m promoters. The BRE2m promoter resembles a coincidence detector (‘logical AND gate’): Mono-stimulation with either BMP or IL6 raises luciferase activity to intermediate levels only, and co-stimulation with both ligands is required for maximal expression (Fig. 1C). This suggests that BRE1 and BRE2 fulfill very distinct functions in hepcidin regulation: The BRE2m promoter shows only a slight reduction in basal luciferase activity compared to the WT promoter. This reduction in basal activity did not reach statistical significance (paired t-test), and was much less pronounced than the effect of a BRE1 mutation. The promoter responsiveness to IL6 mono-stimulation was significantly reduced by the BRE1 mutation but not by the BRE2 mutation (paired t-test). Thus, BRE2 has lesser impact on the IL6-induced fold-change in hepcidin expression than BRE1. The most prominent feature of the BRE2m promoter is the reduced ability to respond to BMP stimuli compared to the WT promoter (0.001<p<0.0025, paired t-test): maximal BMP stimulation enhances luciferase activity by only 80-fold in BRE2m, when compared to 400-fold in WT and BRE1m. The loss of BRE1 hardly affects the promoter responsiveness to maximal BMP doses, although it has some impact at intermediate BMP doses. Taken together, these data raise the interesting question of how two transcription factor binding sites with very similar sequence can show qualitatively distinct behavior in hepcidin expression regulation.

### Modeling suggests that signaling crosstalk plays a minor role in hepcidin expression regulation

The coordinated regulation of hepcidin expression by BMP and IL6 may involve crosstalk at the level of signal transduction. We analyzed signaling pathway interactions by measuring the phosphorylation of STAT3 and SMAD1/5/8 after stimulation with BMP and/or IL6 using quantitative immunoblotting. Cells were stimulated for 12 h after starvation, and phosphorylated SMAD1/5/8 and STAT3 were detected in the cell lysate using phospho-specific antibodies (Supplemental Fig. S2). The signals were quantified by densitometry and the results of biological duplicates were merged (see Methods). SMAD1/5/8 but not STAT3 showed basal phosphorylation in unstimulated cells which is consistent the role of BRE1 in controlling basal luciferase activities (Fig. 1C). As expected, stimulation with BMP or IL6 alone resulted in dose-dependent increases of SMAD1/5/8 and STAT3 phosphorylation, respectively (Fig. 1D). Co-stimulation with a saturating dose of IL6 appeared to slightly reduce BMP-induced SMAD1/5/8 phosphorylation (Fig. 1D, bottom), but the effect is not statistically significant (paired t-test). High doses of BMP had significant inhibitory effects on IL6-mediated STAT3 phosphorylation (p<0.001, paired t-test). However, the effect was moderate and typically resulted in a less-than two-fold reduction in phospho-STAT3 levels (Fig. 1D, top). Thus, while co-stimulation with BMP and IL6 enhances hepcidin expression relative to mono-stimulation, a slight cross-inhibition is observed at the level of transcription factor phosphorylation. We integrated these measurements at different levels into a mathematical model to quantitatively understand the determinants of hepcidin expression regulation.

Our model describes luciferase activity as a function of the extracellular IL6 and BMP concentrations, and consists of two modules: The signaling module describes SMAD and STAT TF phosphorylation in response to BMP and IL6 stimulation, while the promoter module characterizes combinatorial TF binding to the promoter and gene expression. The kinetic parameters of the model as well as the regulatory details at the promoter level were unknown. We therefore estimated the parameters by model fitting to TF phosphorylation and luciferase activity data (Figs. 1C and D), and systematically compared the ability of different promoter variants to fit the data in an unbiased modeling approach.

The signaling module assumes that the dose-response curves of IL6-induced STAT phosphorylation and BMP-induced SMAD phosphorylation are of sigmoidal shape, as suggested by previous studies [4], [40]. Dose-response curves of TF phosphorylation are therefore represented using sigmoidal Hill equations (Supplemental Text S3). Inhibitory crosstalk between SMAD and STAT proteins was modeled by assuming that the phosphorylation degree of one TF affects the Hill equation parameters describing the other pathway.

**Figure 2:**
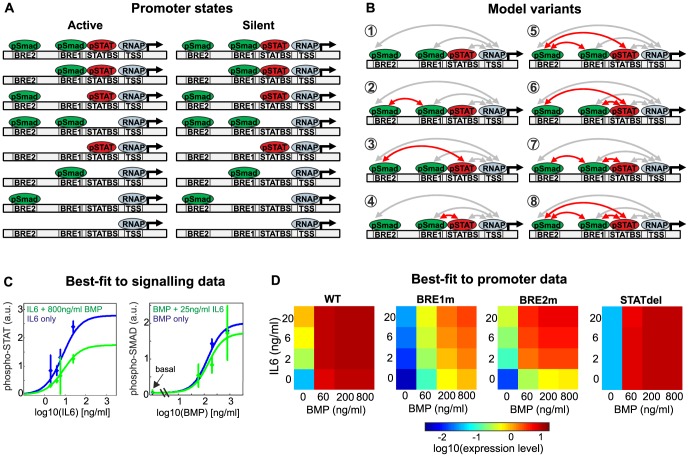
Mathematical modeling of signaling and promoter crosstalk. (**A**) Thermodynamic modeling of promoter states. Depending on the transcription factor concentrations, the hepcidin promoter may be occupied by pSMAD (bound to BRE1 or BRE2), pSTAT (bound to STATBS) and RNAP (bound to the transcription start site), alone or in combination, giving rise to 16 different promoter states. A central presumption of thermodynamic modeling is that all RNAP-bound states are capable of transcription initiation, while RNAP-less states are considered silent. (**B**) A model selection approach allows for the identification of protein-protein interactions on the promoter. Various model variants were tested for their ability to fit the data in Fig. 1C. The minimal model (model 1) assumes that each transcription factor independently activates RNAP (grey arrows), while more complex variants additionally take cooperativity among transcription factors into account (red arrows). Statistical criteria (Akaike information criterion, likelihood ratio test) indicate that model topology 4 is best suited to describe all data (see Methods, Supplemental Protocol S2). (**C**) and (**D**) Integrative crosstalk model simultaneously fits luciferase data and dose-response curves of transcription factor phosphorylation. The thermodynamic promoter model (topology 4 in panel B) was coupled to a simple signaling model describing inhibitory crosstalk between phospho-SMAD and phospo-STAT transcription factors (Supplemental Protocol S2). Solid lines in C represent model trajectories in comparison to experimentally measured data points (shown as mean +/− std). The simulated luciferase activities in D agree well with the corresponding data in Fig. 1C.

The thermodynamic promoter model describes luciferase activity as a function of the phosphorylated TF concentrations, and assumes that TFs affect the occupancy of the promoter: If the pSMAD and pSTAT concentrations are zero, the promoter will either be completely empty or RNA Polymerase II (RNAP) may bind to the transcription start site (TSS) at a basal level (Fig. 2A, bottom row). For increasing TF concentrations the promoter will be occupied by pSMAD, pSTAT or RNAP, or a combination of these, giving rise to multiple promoter states. The presence of three specific binding TF sites and RNAP binding to the TSS yields to 2^4^ = 16 promoter states (Fig. 2A). In which of these states the promoter exists depends in a complex manner on the concentration of phosphorylated TFs, their binding affinity for DNA and may also be altered by TF/TF or TF/RNAP interactions on the promoter. Equations that describe the probability of each promoter state as a function of protein concentrations and binding affinities can be derived based on principles of statistical thermodynamics (Supplemental Text S2, Supplemental Protocol S2).

The promoter states in the model are linked to gene expression by assuming that RNAP-bound promoters are transcriptionally active, while promoters devoid of RNAP binding are silent (Fig. 2A). A transcription initiation rate can thus be calculated from the probabilities of the promoter states (Supplemental Text S2, Supplemental Protocol S2). We neglected gene regulation at the levels of transcription elongation and post-transcriptional processing in our model. The experimentally measured luciferase activity is therefore assumed to be proportional to the simulated initiation rate.

All variants of the promoter module comprised well-known aspects of promoter regulation such as pSTAT/pSMAD binding to the promoter and RNAP recruitment by TFs (grey arrows in Fig. 2B). We additionally allowed cooperative TF binding, implying that TFs may mutually enhance their recruitment to the promoter (red arrows in Fig. 2B). Given three possible cooperative TF interactions alone or in combination, we considered 8 promoter module variants in total (Fig. 2B). Model fitting was done by minimizing the χ^2^ metric which allows for larger difference between model and experiment if the experimental error is large (see Methods). Model variants with different numbers of parameters were compared with respect to goodness-of-fit to the training data using the Akaike information criterion (see Methods). Based on these measures, the data were best explained by a promoter module containing a single cooperative interaction among SMAD and STAT TFs bound to BRE1 and STATBS, respectively (model 4 in Fig. 2B). More complex models also containing the cooperative interaction between BRE1 and STATBS (models 6—8 in Fig. 2B) did not fit the data better than the selected model. Model variants lacking the BRE1-STATBS cooperativity (models 1—3 and 5 in Fig. 2B) fitted the experimental data less well than the selected model from a quantitative point of view. Moreover, they qualitatively failed to explain why IL6 mono-stimulation induces a lesser fold-change in the BRE1m construct when compared to WT (Supplemental Fig. S3, Fig. 1C, [19]).

**Figure 3:**
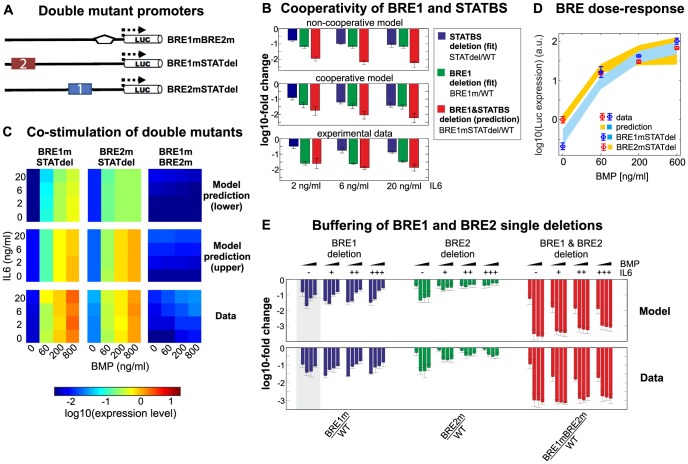
Verification of model predictions using double-mutant promoters. (**A**) Schematic representation of double-mutant promoters which lack two transcription factor binding sites (cf. Fig. 1B). (**B**) Systematic analysis of transcription factor binding site deletion effects confirms cooperativity of BRE1 and STATBS. The impact of binding site deletions was calculated by taking the luciferase activity ratios of different promoters (indicated in the legend) and expressed as a log10-fold change (y axis). As expected for a system where both sites cooperatively enhance transcription, the fold-change upon a combined deletion of BRE1 and STATBS (red) is less than the product of the single deletion fold-changes (green and blue; see text). Data points are mean and standard deviation, and model predictions represent the range of measurement-compliant parameter sets, as derived from a parameter identifiability analysis (see Methods, Supplemental Protocol S2). Only BRE1 and STATBS (but not BRE2) contribute to expression upon IL6 stimulation. (**C**) and (**D**) Co-stimulation heatmaps of double mutant promoters reveal that BRE1 and BRE2 are functionally similar in the absence of STATBS. (C) Heatmaps of luciferase activity under co-stimulation conditions. (D) Two-dimensional projection of the BRE1mSTATdel and BRE2mSTATdel data in C (averaged over all IL6 concentrations). Data points are mean (panel C, bottom row) or mean +/− std (panel D) (n = 6). Model predictions were formulated as ranges based on a parameter identifiability analysis (see Methods, Supplemental Protocol S2), and show measurement-compliant parameter sets with highest and lowest predicted luciferase expression (top and middle rows in panel C; edges of shaded corridors in panel D). Data and model in D were normalized to basal luciferase expression in the BRE2mSTATdel construct. (**E**) Systematic analysis of transcription factor binding site deletion effects confirms buffering of BRE1 and BRE2 single deletions. Concepts similar to panel B. The fold-change upon a combined deletion of BRE1 and BRE2 (red bars) is higher than the product of the single deletion fold-changes (green and blue bars; see text). BMP stimulation conditions were considered to ensure that BRE1 and BRE2 both contribute to expression.

The best-fit model (Figs. 2C and D) comprises 19 kinetic parameters and describes the data with an accuracy close to experimental measurement noise (χ^2^ = 124, N = 80). Nine model parameters enter the sigmoidal Hill functions which describe the stimulus-induced TF phosphorylation, and signaling crosstalk between transcription factors. The remaining 10 parameters enter the promoter module, and describe the TF affinity for DNA binding sites, the TF interaction strength with RNAP, and the cooperativity of TF binding to DNA. The nature of the model parameters is described in detail in Supplemental Protocol S2, and a list of best-fit parameter values can be found in Supplemental Table S1. Not all model parameters could be unequivocally identified based on the experimental data, implying that multiple parameter sets yield a comparable fit to the training data (Supplemental Fig. S4). This non-identifiability of parameters gives rise to uncertainties in the model predictions. We therefore performed all model analyses for many measurement-compliant parameter sets (with χ^2^<135), not only for the best-fit solution (see Methods). The model predictions in Fig. 3 were therefore formulated as a range corresponding to the simulation runs with the highest and lowest predicted luciferase activity. In most cases, reliable model predictions were possible despite non-identifiability of individual parameter values.

In our model, hepcidin expression was mostly determined by the dynamics of TF binding to the promoter, while inhibitory signaling crosstalk played only a minor role: Elimination of signaling crosstalk did not significantly change the simulated luciferase activities, and this conclusion held true for all measurement-compliant parameter sets (Supplemental Fig. S5). Moreover, we compared the ability of model variants with and without signaling crosstalk to fit the luciferase activity data (Fig. 1C). The fit of the crosstalk-less model to the training data was significantly better as judged by the Akaike information criterion (Supplemental Fig. S5). We therefore focused our model validation on regulation events at the promoter level, and neglected the relatively weak signaling crosstalk effects.

### Cooperativity and competition at the promoter shape the hepcidin expression response

We sought to verify our model by an independent set of experiments not used for model calibration. The formulation of model predictions was focused on double mutant promoters which simultaneously lack two TF binding sites (Fig. 3A).

One central promoter mechanism predicted by the model is the cooperative interaction between pSMAD and pSTAT TFs, bound to BRE1 and STATBS, respectively. The double mutant promoter lacking functional BRE1 and STAT elements (BRE1mSTATdel promoter, Fig. 3A) was employed to independently confirm the cooperativity effect. The BRE1STATdel promoter shows a ∼30-fold reduced expression relative to WT upon stimulation with 2 ng/ml IL6 (Fig. 3B, red bar). The corresponding STATBS and BRE1 single deletions reduce expression by ∼3 and ∼30-fold, respectively (Fig. 3B, blue and green bars). Thus, the BRE1m and BRE1mSTATdel promoters exhibit similar expression levels. This is consistent with BRE1-STATBS cooperativity, because the single BRE1m single deletion already eliminates the cooperativity effect, and thereby a large part of the STATBS contribution to expression. In contrast, a transcription model lacking the cooperativity would predict an independent contribution of both sites, implying that the expression reduction in the BRE1mSTATdel promoter equals the product of the single deletion fold-changes (Fig. 3B, top). Thus, the double mutant data qualitatively supports the model with cooperativity between BRE1 and STATBS, also for higher doses of IL6 (Fig. 3B).

BRE1 and BRE2 play different roles in hepcidin expression with respect to basal expression, BMP inducibility and co-stimulation response. One difference between the two sites is the above-mentioned cooperative interaction with the STATBS that is specific to BRE1. How do the BMP-responsive elements differ beyond this interaction? The model predicted that BRE1 has higher affinity for phosphorylated SMAD than BRE2, explaining why BRE1 plays a predominant role under basal conditions. Upon sufficiently strong BMP stimulation both sites are predicted to activate RNAP with comparable efficiency. In conclusion, the model suggested that BRE1 and BRE2 should behave similarly in the absence of cooperative promoter interactions. This prediction can be tested by co-stimulation of BRE2mSTATdel and BRE1mSTATdel promoters which solely contain BRE1 and BRE2, respectively (Fig. 3A). The experimental data was in good qualitative agreement with model predictions: Both mutants showed very similar co-stimulation heatmaps and primarily responded to BMP stimulation (Fig. 3C, bottom row). Maximal luciferase activity at high BMP levels was comparable for both constructs, indicating that BRE1 and BRE2 indeed drive RNAP activation with similar efficiency (Figs. 3C and D). Basal activity was approximately 10-fold higher in the BRE2STATdel promoter, suggesting that the isolated BRE1 has indeed a higher pSMAD affinity than BRE2 (Fig. 3D). Quantitative model predictions for the BRE2mSTATdel and BRE1mSTATdel heatmaps were only possible up to a certain range of absolute luciferase activities owing to non-identifiability of model parameters (Fig. 3C, top and middle row). The experimentally observed luciferase activities were within the predicted range (Fig. 3C).

The model suggested that saturating RNAP binding to the TSS limits hepcidin expression upon strong stimulation, whereas signaling pathway saturation plays only a minor role. Saturation implies that single deletions promoters should exhibit expression levels relatively similar to WT, because the remaining TF binding sites maintain near-complete occupancy of the TSS with RNAP. This buffering effect should be abrogated upon a combined TF binding site deletion, leading to the prediction that hepcidin double mutant promoters exhibit very low expression compared to the corresponding single mutants.

We confirmed this prediction by measuring the activity of the BRE1mBRE2m double mutant promoter (Fig. 3E): The double deletion of BRE1 and BRE2 reduces the luciferase activity by up to ∼1000-fold compared to WT (red bars). The corresponding single deletions typically reduce expression by ∼10-fold or less, and are thus much more similar to the WT promoter (blue and green bars).

This behavior indicates TSS saturation, because the fold-change by a double deletion equals the product of the corresponding single deletion fold-changes in thermodynamic transcription models without saturation. The validity of our model is further supported by the quantitative agreement of the models' predictions with the data: the buffering of single deletions is more pronounced for strong (co-) stimulation (Fig. 3E), where promoter saturation is particularly prominent. We find similar, albeit less pronounced, buffering effects between STATBS and BRE2 (Supplemental Fig. S6) and conclude that saturating RNAP binding is an important aspect of promoter behavior.

Promoter saturation also explains why the BRE1 single mutation strongly reduces expression at intermediate BMP concentrations, while having lesser impact at basal and strong stimulation conditions (Fig. 3E, blue bars, grey corridor). The BRE1 deletion has a strong effect on expression at intermediate BMP doses, because the high-affinity BRE1 is already fully occupied by pSMAD. Higher BMP concentrations alleviate the impact of BRE1 deletions, because low-affinity pSMAD binding to the BRE2 saturates the promoter, thereby buffering the loss of BRE1.

### The steepness of hepcidin promoter regulation plays a key role for systemic iron homeostasis

**Figure 4:**
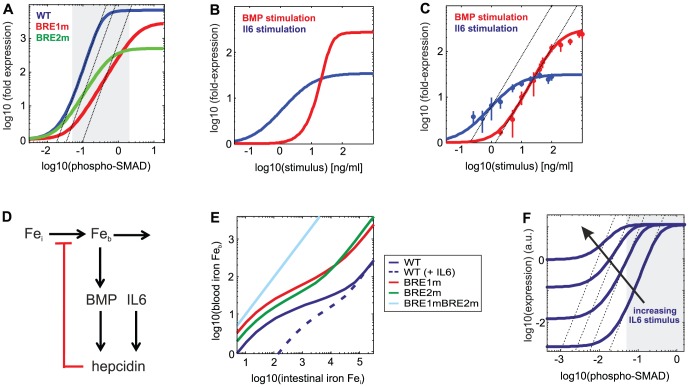
Systems properties of hepcidin expression. (**A**) The presence of two BREs enhances promoter sensitivity towards BMP stimulation. Hepcidin expression (fold over basal) is shown as a function phospho-SMAD levels for the WT, BRE1m, and BRE2m promoter (phospho-STAT was assumed zero). The dashed lines indicate the maximal steepness of the WT dose-response. The grey corridor indicates range of phospho-SMAD levels in HuH7 cells. (**B**) and (**C**) Hepcidin expression is highly sensitive to BMP stimulation, and less sensitive to IL6. The luciferase activity (fold over basal) is plotted as function of the IL6 (blue) or BMP (red) concentration. Panel B shows simulations of the best-fit model, while panel C contains experimental data (n = 3–6) and fits of the Hill equation (solid lines). Dashed lines in C indicate the maximal steepness of the BMP response. (**D**) Extended mathematical model describing negative feedback regulation of iron blood levels by hepcidin *in vivo*. Iron blood levels (Fe_b_) are controlled by influx and efflux reactions, and the iron influx rate is proportional to the intestinal iron concentration (species Fe_i_). Iron blood levels control the BMP signaling pathway, and thus the expression of hepcidin, which in turn lowers the iron influx. Hepcidin expression regulation by IL6 and BMP was modeled using the best-fit crosstalk model (Fig. 2C and D; Supplemental Text S4). (**E**) Iron homeostasis requires two BMP-responsive elements and is abolished by inflammatory stimulation. Simulations of the extended model (panel D) show how iron blood levels respond to changes in the intestinal iron concentration. The model with a WT hepcidin promoter (blue solid line) shows relatively constant iron blood levels over a broad range of intestinal iron concentrations (‘homeostasis range’). Homeostasis is less efficient and the homeostasis range is narrower in model variants with BRE1m and BRE2m promoters, or if strong IL6 stimulation is assumed (see legend) The mutants are characterized by altered iron blood levels (reflecting iron overload and deficiency, respectively). (**F**) IL6 stimulation reduces the BMP sensitivity of the promoter. The best-fit model (Fig. 2C and D) was employed to simulate how increasing IL6 stimulation affects the BMP dose-response curve of the promoter. Dashed lines indicate the maximal slope in the absence of IL6. Grey corridor same as in A.

We investigated *in silico* how the simultaneous presence of two BREs affects the behavior of the hepcidin promoter, and found that this promoter design enhances the steepness of the BMP dose-response curve: A certain increase in phospho-SMAD levels induces a larger expression fold-change in the WT promoter when compared to BRE1m and BRE2m promoters (Fig. 4A). The hepcidin promoter only contains a single STAT binding site as opposed to two BREs. Accordingly, the model predicts that the hepcidin promoter specifically responds with high sensitivity towards BMP stimulation, and is much less sensitive towards IL6 stimulation (Fig. 4B). The initial stimulation experiments used for model training (Fig. 1C) were based on three BMP and IL6 concentrations, and therefore did not allow for conclusions concerning the steepness of the BMP and IL6 response. To verify the model prediction by an independent set of experiments, we performed detailed dose-response measurements with multiple doses of IL6 and BMP, respectively (Fig. 4C). These data confirm that the BMP response is much steeper than the IL6 response, and thereby validate the model.

Systemic iron homeostasis is maintained by an auto-regulatory negative feedback loop in which hepcidin expression levels depend on and control the circulating iron levels in the blood (Fig. 4D). A high BMP sensitivity of the promoter may allow the iron-BMP signaling axis to sense minor changes in iron blood levels, and to maintain systemic iron homeostasis. We simulated iron homeostasis in the living animal using an extended model with feedback (Fig. 4D). Iron blood levels were described by the species Fe_b_, whose levels are controlled by influx and efflux reactions. The iron influx rate is proportional to the intestinal iron concentration (species Fe_i_) which reflects the dietary iron content. Iron blood levels control the activity of the BMP signaling pathway, and thus hepcidin expression. Negative feedback regulation was considered in the model by assuming that the iron influx is negatively influenced by hepcidin (Supplemental Text S4).

Hepcidin expression regulation by BMP and IL6 in the model was described using the previously derived best-fit model (Figs. 2C and D). The remaining parameters of the model describe the iron influx and efflux, hepcidin degradation, and the strength of hepcidin-mediated feedback on the iron influx.

Homeostasis was analyzed by assessing how the iron blood level in the model (Fe_b_) responds to a change dietary iron content (Fe_i_), efficient homeostasis implying that a given fold-change in Fe_i_ elicits a much lesser fold-change in Fe_b_. The key assumption we made was that hepcidin-mediated feedback regulation has a very strong impact on the iron influx. For the limit of strong feedback, it can be shown analytically that the degree of iron homeostasis is solely determined by the steepness of the hepcidin promoter response, and independent of the remaining model parameters (Supplemental Text S1). The range of intestinal iron concentrations for which homeostasis is observed is determined by the range of BMP concentrations that can be sensed by the hepcidin promoter (Supplemental Text S1). The model thus allowed us to quantitatively analyze how the hepcidin promoter architecture affects systemic iron homeostasis, although the remaining model parameters were unknown.

The simulations in Fig. 4E show that a model with the WT hepcidin promoter efficiently maintains systemic iron homeostasis, as the iron blood levels remain essentially constant over a broad range of intestinal iron concentrations. Models with BRE1m and BRE2m promoters perform less well, as the perturbation-response curves are steeper and homeostasis is restricted to a narrower range of influx rates (Fig. 4E, green and red curves). This suggests that the simultaneous presence of two BMP-responsive elements in the promoter indeed optimizes the performance of the systemic iron homeostasis loop.

One important question is why IL6 stimulation reduces iron blood levels and induces anemia of inflammation even though the homeostasis loop should effectively buffer IL6-induced perturbations in hepcidin expression. Simulations of the extended feedback model show strongly diminished iron levels and a loss of homeostasis if high IL6 levels are assumed (Fig. 4E, blue dashed line). This effect can be understood by considering the BMP dose-response curve of the best-fit promoter model for varying IL6 concentrations (Fig. 4F): increasing IL6 levels reduce the sensitivity of the BMP dose-response curve due to (partial) saturation of the TSS with RNAP. Moreover, significant changes in hepcidin expression are restricted to a narrower range of phospho-SMAD levels. The hepcidin promoter therefore responds less efficiently to changes in the iron/BMP signaling in the presence of IL6. This impairs the iron sensing capability of the hepcidin promoter *in vivo*, and leads to a breakdown of feedback homeostasis.

Iron blood levels are chronically elevated in HH, in most cases due to inactivating mutations in the iron-sensing BMP signaling axis. One unexplored question is why HH is commonly associated with inactivating mutations in the SMAD signaling pathway, while mutations in the BRE1 promoter element are rare and BRE2 mutations have not been identified yet [20]. The iron homeostasis model predicts that a BRE1 deletion affects the iron blood levels more strongly than the BRE2 deletion in the WT homeostasis range (compare green and red lines in Fig. 4E, respectively). The more critical role of BRE1 may explain why only BRE1 mutations have been associated with HH, and can be explained by its higher phospho-SMAD affinity when compared to BRE2. The model further predicts strong buffering of BRE1 and BRE2 single deletions: Single mutations in either site have much weaker effects than a complete feedback ablation by a BRE1mBRE2m double mutation (light blue line in Fig. 4E). The very strong effect of a BRE double deletion in cultured cells (Fig. 3E) is thus predicted to be preserved *in vivo*. These simulations may explain why BMP signaling pathway mutations that simultaneously inactivate expression regulation via BRE1 and BRE2 are by far the most common cause of HH.

Taken together, the steepness of the hepcidin promoter response appears to be a key parameter controlling how well the systemic iron homeostasis loop compensates for fluctuations in iron diet content.

## Discussion

The fine-tuned expression of hepcidin plays a central role in systemic iron homeostasis, and is deregulated in two major clinical settings, HH and anemia of inflammation. Here, we comprehensively characterized the gene regulatory function of the hepcidin promoter using systematic promoter mutagenesis and co-stimulation experiments. We employed a multi-scale modeling approach capturing signaling and gene expression, and discriminated various promoter regulation scenarios. This approach complements existing strategies linking signaling and gene expression events [41], [42], and may be extended in future studies to model global transcription patterns based on mRNA expression profiles, TF binding information and mRNA half-life data.

Gene expression may be a gradual or a binary event at the single cell level [43]. Our mathematical model assumes that TF phosphorylation and reporter gene expression measurements in a cellular ensemble reflect the behavior of single cells, and thus presumes a gradual mode of hepcidin expression. Experimental studies indicate that BMP-induced target gene expression is indeed a gradual event at the single-cell level [4]. In any case, the population-based model reflects physiologically relevant aspects of hepcidin expression, because systemic hepcidin levels *in vivo* are governed by expression in an ensemble of hepatocytes.

The architecture of the hepcidin promoter was characterized in detail in previous studies, and BRE1, BRE2 as well as STATBS were identified as central *cis*-regulatory elements mediating BMP and IL6 responsiveness [19], [24], [44]. Our results confirm the central role of the STATBS, as STATBS deleted promoters cannot be induced by IL6 stimulation. The critical role of BRE1 and BRE2 for the BMP responsiveness was shown by reporter gene assays with truncated versions of the promoter, and is also supported by the high sequence conservation of these elements [17]–[19], [44], [45]. In a search for additional BREs, we performed a linker scanning analysis of the hepcidin promoter, systematically replacing short nucleotide stretches along the promoter sequence (unpublished data). This analysis revealed no additional BMP target motifs. Accordingly, we observe that the BRE1mBRE2m double mutant shows near complete ablation of basal expression and BMP responsiveness (Fig. 3C). However, stimulation with very high doses of BMP appears to slightly enhance expression from the BRE1mBRE2m promoter (Fig. 3C). We suggest that highly active BMP receptors may weakly phosphorylate SMAD2/3 TFs which are part of the TGFβ signaling pathway, thereby activating the previously described TGFβ-responsive elements in the hepcidin promoter [46].

Our model suggests that BRE2 enhances transcription with similar or higher efficiency than BRE1 (Fig. 3, Supplemental Fig. S4), thus raising the question of how a sequence element located as far as 2 kb away from the TSS enhances transcription with high efficiency. The 2 kb distance from the TSS corresponds to a length of 680 nm along the strand; this number exceeds the length of the mediator complex (∼40 nm) which links RNA polymerase to transcription factors [47]. Thus, BRE2-mediated transcription initiation likely involves DNA looping. Our current model neglects the details of DNA looping, but could be extended by existing quantitative modeling approaches taking into account the thermodynamics of DNA bending [48], [49]. Such a detailed promoter model should also consider that the BRE2 element of the hepcidin promoter is flanked by bZIP, HNF4alpha/COUP binding sites in the immediate neighborhood [17]. The single deletion of the bZIP or HNF4alpha/COUP sites reduces the BMP responsiveness of the hepcidin promoter. This indicates that a complex of multiple transcription factors cooperates at the BRE2 site to recruit RNAP which may explain the apparently high efficiency of BRE2 in driving transcription. Another prediction of our model is that the strong impact of the BRE1 in the basal state is due to its high affinity for phospho-SMAD binding when compared to BRE2 (Supplemental Fig. S4, Fig. 3E). Both BREs are characterized by the same sequence motif (GGCGCC), suggesting that epigenetic differences in the chromatin state may be responsible for the apparently different affinity of BRE1 and BRE2. Taken together, the present model is likely to be a simplified representation of the real events at the promoter. Future studies are required to model individual events which are currently lumped into overall interaction energies.

Different modes of signal integration may be realized in transcriptional regulation. Two stimuli may control expression in an additive or multiplicative way. We systematically compared the co-stimulation response of the hepcidin promoter with the corresponding mono-stimulation responses (Supplemental Fig. S7). The fold-change in expression upon co-stimulation is generally less than the product of the mono-stimulation fold-changes in the WT promoter (Supplemental Fig. S7; Fig. 1C), as also supported by previous studies in HuH7 and Hep3B cells [50], [51]. Analytical studies in the Supplemental Text S1 show that this sub-multiplicative signal integration explains why IL6 co-stimulation leads to a breakdown of systemic iron homeostasis (Fig. 4E): Homeostasis is lost, because IL6 reduces the BMP-induced fold-change in expression, thereby reducing the efficiency of negative feedback regulation. Our model suggests that the less-than multiplicative behavior of the WT promoter arises from saturating RNAP binding to the TSS. The saturation effect is less pronounced in single mutant promoters, explaining why these exhibit near-multiplicative signaling integration (fold-change over basal in response to co-stimulation equals the product of the mono-stimulation fold-changes) (Supplemental Fig. S7). Interestingly, the model predicts and experiments support that pSMAD and pSTAT may also drive hepcidin expression in a synergistic, more-than multiplicative manner due to the presence of the cooperative BRE1/STATBS interaction if promoter saturation effects are negligible: We changed the basal pSMAD level in the model, and observed that a certain basal BMP signaling activity is required for optimal responsiveness of the promoter towards IL6 mono-stimulation (Supplemental Fig. S7). This model prediction is supported by experiments in HuH7 cells showing that SMAD4 siRNA lowers the IL6 inducibility of the hepcidin promoter ([46]; unpublished observation), and by data in hepatocyte-specific SMAD4 knockout mice [52]. We conclude that the hepcidin promoter shows high plasticity in the integration of BMP and IL6 signals, depending on the strength of basal and induced signaling.

In this study, we used *in vitro* measurements in HuH7 cells to parameterize an *in vivo* model of systemic iron homeostasis, thereby assuming that HuH7 cells quantitatively reflect hepcidin regulation *in vivo*. HuH7 cells have been widely used in the field of iron metabolism to study hepcidin regulation, and have been to behave very similarly to other hepatoma cells (HepG2, Hep3B) and primary hepatocytes [17], [19], [23], [44], [46], [50], [53]. While, to the best of our knowledge, there are no reports of quantitative comparisons on hepcidin regulation *in vivo* and *in vitro*, there is abundant evidence that the *in vivo* data that qualitatively mirrors the results obtained *in vitro*. For instance, Wang et al. reported that SMAD4 is essential for hepcidin activation both in mice and in primary hepatocytes [52], while Pietrangelo et al. showed in both models that STAT3 is a key transcription factor for IL-6 activation of hepcidin gene expression [54]. These results can be consistently reproduced in HuH7 cells, suggesting that the molecular mechanisms involved in these signaling pathways are preserved in this cell line.

The hepcidin promoter contains two BMP-responsive elements as opposed to a single STAT binding site, raising the question of why such a promoter design may be advantageous for the regulation of systemic iron homeostasis. We find that the presence of two BREs ensures that hepcidin expression is very sensitive towards changes in the iron-sensing BMP pathway (Fig. 4B–D). This makes the negative auto-regulation loop more nonlinear, thereby promoting systemic iron homeostasis (Fig. 4E). Systems biology studies at the organismal level are required to confirm that our simple model of iron homeostasis faithfully predicts the dynamics of iron metabolism *in vivo*.

## Materials and Methods

### Model implementation

The mathematical model describing hepcidin expression (used to generate Figs. 2C, 2D, 3B–3E, 4A–4C and 4F) consists of two modules: (i) the signaling module which describes the phosphorylation of SMAD and STAT transcription factors as a function of the BMP and IL6 concentrations. (ii) the promoter module uses the concentrations of phospho-SMAD/STAT (described by module i) as inputs, and computes the hepcidin expression level as an output. In the signaling module, we described the dose-response behavior of SMAD/STAT phosphorylation at steady state using sigmoidal Hill equations (Supplemental Text S3). Potential signaling crosstalk was considered by assuming that the phosphorylation of one TF modulates the maximal phosphorylation of the other TF (Supplemental Text S3). The promoter module was described based on the thermodynamic model derived in Supplemental Text S2. In Supplemental Protocol S2, we describe how this thermodynamic model was applied to hepcidin expression regulation, combined with the signaling model and we also provide a detailed description of all model parameters. The hepcidin expression model was embedded into an ODE model to describe systemic iron homeostasis by hepcidin-mediated negative feedback (Figs. 4D and E). A detailed description of this ODE model can be found in Supplemental Text S4.

### Model fitting

The models were fitted to the experimental data by minimizing χ^2^ metric, given by χ^2^ = (M_i_-s_j_⋅D_i_)^2^/σ_i_^2^. M_i_, D_i_ and σ_i_ are the simulated value, the measured value and the experimental error, respectively. The fitted scaling factor s_i_ accommodates that the model is formulated in absolute concentration units, while signaling and luciferase activities could only be measured in arbitrary units. The simulated phospho-SMAD/STAT concentrations were fitted to the immunoblotting data (Fig. 2C), while the simulated transcription initiation rate was fitted to the luciferase measurements (Fig. 2D), i.e., gene expression was assumed to be at steady state (see text). Parameter optimization was done using a deterministic trust region optimizer in Matlab. In order to circumvent local minima, we repeatedly fitted the model starting from 80.000 quasi randomly distributed positions in the space of allowed parameter ranges. The optimization apparently converged to a global optimum, because ∼45% of the fitting runs yielded χ^2^ values close to the minimum all 80.000 runs. We fitted model topologies of different complexity by eliminating certain reaction steps (Fig. 2B, Supplemental Protocol S2), and compared the ability of model variants to fit the data. Models of different complexities were compared based on their goodness-of-fit to the training data set using the Akaike Information criterion (AIC = χ^2^+2*k*; *k*…number of model parameters) and the likelihood ratio test (Supplemental Protocol S2). Both statistical measures favored model topology 4 in Fig. 2B.

### Parameter identifiability

Parameter identifiability was analysed using the strategy proposed by Hengl et al. [55]: The parameter vectors of the top 45% fitting results from quasi-randomly distributed starting parameter sets (see above) had a similar goodness-of-fit (χ^2^<135), and were analysed with respect to parameter ranges and parameter correlations (Supplemental Fig. S4). The robustness of model predictions was estimated by repeatedly simulating predictions for the top 45% of the model solutions. The simulations predicting the highest and lowest values are given as a prediction range in Fig. 3.

### Cell culture

The human hepatocarcinoma HuH7 cell line was cultured in Dulbecco's Modified Eagle's Medium (DMEM, high glucose; Invitrogen) supplemented with 10% heat inactivated low-endotoxin fetal bovine serum (FBS, Invitrogen), 1% penicillin, 1% streptomycin and 1 mM Sodium Pyruvate. Cell cultures were maintained in a 5% CO_2_ atmosphere at 37°C.

### Luciferase reporter gene assays

Generation of the luciferase reporter construct containing a 2762-bp fragment of the human hepcidin promoter (WT) and derivate constructs with mutations in BMP-responsive element 1(position −84/−79; BRE1m), BMP-RE2 (position −2255/−2250; BRE2m), STAT binding site (position −72/−64; STATdel), and in BMP-RE1 and BMP-RE2 (BRE1mBRE2m), have been previously described [19], [44]. In this study we generated two additional reporter constructs that combined mutations in BMP-RE1 or BMP-RE2 with the deletion of the STAT binding site (constructs BRE1STATdel and BRE2STATdel, respectively). HuH7 cells (1.5×10^5^ per well) were seeded onto six-well plates. The next day, 500 ng of pGL3 reporter vectors containing the hepcidin promoter constructs were transfected, together with 10 ng of a control plasmid containing the Renilla gene under the control of the CMV promoter. Plasmid transfections were performed using Lipofectamine 2000 (Invitrogen) according to manufacturer's instructions, and medium was replaced by FBS-free medium. Twenty-four hours after transfection, cells were treated with human BMP-6 (50 ng/ml, 24 h) and/or IL-6 (2 ng/ml, 24 h). Cells were harvested in Passive Lysis Buffer (Promega) for measurement of luciferase activity and cellular extracts were analyzed using the Dual-Luciferase-Reporter assay system (Promega) and a Centro LB 960 luminometer (Berthold Technologies).

### Western blot analysis

HUH7 cells (1.5×10^5^ per well) were seeded onto 6-well plates and the day after the culture medium was exchanged to FBS-free medium. After 12 hours the cells were treated with increasing doses of BMP-6 (60; 200; 800 ng/mL; R&D Systems) and/or IL-6 (2; 4; 6; 25 ng/mL; R&D Systems) for 12 hours and then harvested for protein analysis. Cells were washed twice in ice cold Dulbecco's phosphate-buffered saline (PBS). Cell pellets were lysed in ice-cold NET buffer (1% Triton X-100 (v/v), 50 mM Tris-HCl pH 7.4, 150 mM NaCl, 5 mM EDTA, 20 mM NaF, 1 mM Na_3_VO_4_) supplemented with 1× Complete Mini Protease Inhibitor Mixture (Complete Mini,Roche Applied Science). The protein concentration was measured using the BCA (bicinchoninic acid) Protein Assay (Pierce). Protein lysates (15 µg) were subjected to 10% SDS-PAGE and transferred to a nitrocellulose membrane (Whatman) for protein immunodetection using rabbit anti-phospho SMAD 1/5/8 (Cell Signaling #9511), mouse anti-phospho STAT3 (Cell Signaling, #9138) and mouse anti-actin (Sigma Aldrich, A2228). Blots were then incubated with horseradish peroxidase conjugated anti-mouse or anti-rabbit secondary antibodies (Sigma Aldrich) and then subjected to chemiluminescence (Amersham Biosciences, ECL Plus). For the densitometric analysis the resulting bands were digitalized and quantified using the NIH *Image J* software (rsb.info.nih.gov/nih-image/)

### Data processing

Luciferase Reporter Assays were performed in at least four biological replicates. Reporter gene expression was monitored using firefly luciferase, and co-transfection with Renilla luciferase allowed for correction with respect to cell number and transfection efficacy. The relative light units of firefly luminescence of each experimental condition were divided by the relative light units of the corresponding Renilla luminescence. Each replicate measurement series was normalized by the median over all data points of that series to correct for slight differences in absolute luciferase signals between replicate experiments. Experimental errors were estimated by calculating standard deviations over all replicates. Errors in the fold changes of luciferase expression (Fig. 3B and E) were estimated using a Monte-Carlo approach: Random realizations were drawn from normal distributions with mean and standard deviation equal to those of the measured luciferase expression data. Fold-changes were calculated for 10^3^ pairs of realizations, and the fold-change error was evaluated by calculating the standard deviation of the resulting probability distributions.

Signaling crosstalk was monitored by immunoblotting against phosphorylated SMAD and STAT in two biological replicates (Supplemental Fig. S4). Bands were quantified by densitometry, and duplicate measurements were merged by multiplying one of the duplicate series with a fitted scaling factor to correct for differences in arbitrary units between gels. Figs. 1D and 2C show mean and standard deviation of merged duplicate experiments. Some experimental errors estimated from scaling were unreasonably small; therefore a minimal experimental error was assumed, based on typical variability in Western Blot measurements (relative error of 5% plus an absolute error value).

## Supporting Information

Figure S1 Luciferase measurements reflect endogenous hepcidin mRNA expression. Expression fold-changes in the luciferase assay data (x axis) are strongly correlated with expression fold-changes in qPCR measurements of endogenous hepcidin mRNA (y axis). The green and blue data points indicate qPCR measurements for two independent biological replicates, each with technical replicates (n = 2). The data points correspond to the following stimulus concentrations: 6 ng/ml IL6, 25 ng/ml IL6, 200 ng/ml BMP6, 800 ng/ml BMP6, 200 ng/ml BMP6 + 25 ng/ml IL6, 800 ng/ml BMP6 + 25 ng/ml IL6, and 800 ng/ml BMP6 + 6 ng/ml IL6. The blue and green solid lines show linear fits to the data, and R indicates the Pearson correlation coefficient of each qPCR replicate series. See Supplemental Protocol S1 for a detailed description, and for the qPCR protocol.
(JPG)

Figure S2 Immunoblotting of SMAD/STAT phosphorylation upon co-stimulation indicates moderate inhibitory signaling crosstalk. (**A**)–(**D**) HuH7 cells were stimulated with increasing doses of IL6 in the presence or absence of BMP (A, C) or vice versa (B, D). Signaling crosstalk was analyzed by immunoblotting against phosphorylated SMAD and STAT. Actin levels serve as loading controls. Two biological replicates were performed (Replicate 1: panels A and B; Replicate 2: panels C and D).
(JPG)

Figure S3 Fitting and analysis of a model with non-cooperative STAT and SMAD binding to STATBS and BRE1 sites. (A) and (B) Best-fit of the non-cooperative model (variant 1 in Fig. 2B) with inhibitory signaling crosstalk to luciferase data and dose-response curves of transcription factor phosphorylation (Supplemental Protocol S2). The simulated luciferase activities in A can be compared to the corresponding experimental data in Fig. 1C. Solid lines in B represent model trajectories in comparison to experimentally measured data points (shown as mean +/− std). (C) The non-cooperative model fails to explain the loss of IL6 sensitivity in the BRE1m promoter. Shown are the luciferase heatmaps of WT and BRE1m promoters (rows), as measured experimentally (left column) or simulated using cooperative and non-cooperative models, respectively (middle and right columns). Each heatmap was normalized to the corresponding basal expression level. The BRE1m promoter shows lower IL6 inducibility than WT in the data and in the cooperative model, but not in the non-cooperative model (indicated by green arrow).
(JPG)

Figure S4 Analysis of parameter identifiability. (A) Box plots of the measurement-compliant parameter ranges. The model with inhibitory signaling crosstalk and BRE1-STATBS cooperativity (variant 4 in Fig. 2B) was analyzed, and parameter combinations with a similar goodness-of-fit (χ^2^<135) were collected (see Methods). The box plot indicate the distribution of each parameter (mid line: median; box edges: upper and lower quartile; whiskers contain 1.5 interquartile ranges from the edges; red crosses: outliers). (B) and (C) Relationship of model parameters describing the activities of BRE1 and BRE2. (B) Comparison pSMAD binding affinities of BRE1 and BRE2 (K_B1_ and K_B2_, respectively). (C) Comparison of RNAP interaction strength of BRE1-bound and BRE2-bound pSMAD (f_B1_ and f_B2_, respectively). Each circle corresponds to one measurement-compliant parameter (defined as in panel A), the solid line indicates the bisectrix.
(JPG)

Figure S5 The luciferase measurements can be quantitatively modeled without assuming crosstalk between signaling pathways. (A) Best-fit of a hecipidin expression model without crosstalk at the level of BMP and IL6 signaling pathways. Luciferase expression was simulated using Eqs. S3.1 and S2.13 (Supplemental Text S3 and Supplemental Protocol S2), and the transcription rate in the model (p_bound_) was fitted to the data in Fig. 1C (using a scaling factor). The best-fit parameter values of this model are given in Supplemental Table S1. (B) Removal of signaling crosstalk does not appreciably affect the simulated luciferase activities in fits of the full model. The full model with signaling crosstalk was fitted to the data from multiple starting parameter sets, and all fitting solutions with a comparable goodness-of-fit (χ^2^<135) were analyzed (see Methods): The simulated luciferase levels with signaling crosstalk were plotted against the luciferase activities of model variants where crosstalk was eliminated (setting k_C,1_ and k_C,2_ in Eq. S3.1 to zero). The luciferase activities are essentially unaffected by the deletion of crosstalk.
(JPG)

Figure S6 Buffering of BRE2 and STATBS single deletions. Systematic analysis of transcription factor binding site deletion effects confirms buffering of BRE2 and STATBS single deletions. The impact of binding site deletions was calculated by taking the luciferase activity ratios of different promoters (indicated at the bottom) and expressed as a log10-fold change (y axis). The fold-change upon a combined deletion of BRE2 and STATBS (red) is higher than the product of the single deletion fold-changes (green and blue) at high BMP stimulation. This indicates promoter saturation (see main text). Data points are mean and standard deviation, and model predictions represent the range of measurement-compliant parameter sets, as derived from a parameter identifiability analysis (see Methods). Co-stimulation conditions were considered to ensure that BRE2 and STATBS both contribute to expression.
(JPG)

Figure S7 Integration of BMP and IL6 signals at the level of hepcidin expression. (A) The WT hepcidin promoter integrates BMP and IL6 signals in a sub-multiplicative manner, while mutants show multiplicative behavior. The x dimension shows the experimentally observed fold-expression-change over basal upon co-stimulation with BMP and IL6. The y dimension shows the product mono-stimulation responses over basal with the same doses of BMP and IL6, respectively. Each data point represents one co-stimulation condition (different concentrations of BMP and IL6 and/or different promoter constructs). The colors of the data points correspond to different promoter constructs (legend). The bisectrix (solid line) marks the expectation for a multiplicative system (Co-stimulation fold-change over basal equals the product of the mono-stimulation fold-changes). (B) Basal BMP signaling pathway activity is required for optimal IL6 responsiveness of the hepcidin promoter. The fold expression change in response to very strong IL6 mono-stimulation is shown as a function of the basal phospho-SMAD level (using the parameters of the best-fit WT model). Basal BMP signaling is required for optimal IL6 responsiveness of the promoter, indicating that both stimuli synergistically regulate hepcidin expression (i.e., in a more than multiplicative manner) in this regime of weak BMP signaling. This model prediction is supported by experiments in HuH7 cells showing that SMAD4 siRNA lowers the IL6 inducibility of the hepcidin promoter ([46]; unpublished observation), and with data in hepatocyte-specific SMAD4 knockout mice [52].
(JPG)

File S1 Matlab code of the hepcidin expression model. The file comprises the signaling model describing SMAD and STAT phosphorylation, and the thermodynamic model describing hepcidin promoter regulation. The simulation results (cf. Figs. 2C and D) are plotted together with mean and error in the experimental data (cf. Figs. 1C and D).
(TXT)

Protocol S1 Comparison of luciferase signals with endogenous hepcidin mRNA expression.
(PDF)

Protocol S2 Model implementation, model fitting and model selection.
(PDF)

Table S1 Best-fit model parameters. Best-fit parameters for the models with and without signaling crosstalk (Figs. 2C/D and Supplemental Fig. S5, respectively). The parameters 1–9 belong to the signaling module of the model, while the remaining ones describe promoter regulation. Each parameter was constrained to a physiologically feasible range during fitting. The Hill coefficients of the signaling module (parameters 3 and 7) were restricted to values typical for biochemical response curves. The other parameters of the signaling module (y_max_, EC_50_) represent a combination of multiple signaling reaction constants, and were thus constrained such that they match the experimental measurements of transcription factor phosphorylation (Fig. 1D). Most parameter ranges of the promoter module were taken from the literature. Some were allowed to vary over a broad range to accommodate different kinds of qualitative behavior. For example, the wide range of half-maximal promoter saturation constants (K_P_; parameter 10) allows for promoter saturation to occur upon stimulation. Likewise, the K_D_ values of transcription factor binding to cognate promoter sites (parameters 11–13) were allowed to vary over a broad range to accommodate weak and strong binding. The parameter ranges for the constants describing protein-protein interactions on the promoter (parameters 14–19) represent the typical interaction energies of 1–5 kcal/mol reported in the literature [29]. The exponents n_SM_ and n_ST_ reflect transcription factor dimerization and trimerization, implying that values of up to 3 can be expected.
(PDF)

Text S1 Conceptual model of systemic iron homeostasis.
(PDF)

Text S2 Derivation of a thermodynamic model for the hepcidin promoter.
(PDF)

Text S3 Modeling the dose-response behavior of signaling pathways.
(PDF)

Text S4 Detailed model of systemic iron homeostasis.
(PDF)
