# Supplementary figures and images for: A Multi-Scale Model of Hepcidin Promoter Regulation Reveals Factors Controlling Systemic Iron Homeostasis
Source: PLoS Comput Biol. 2014 Jan 2;10(1):e1003421. doi: 10.1371/journal.pcbi.1003421 (PMC3879105; doi:10.1371/journal.pcbi.1003421)

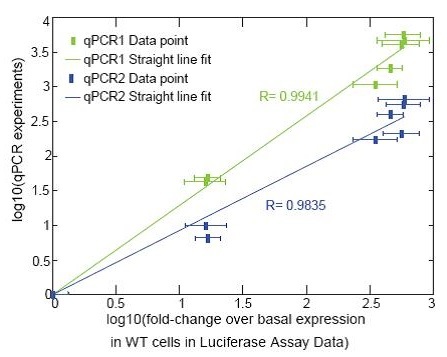

Supplement: Figure S1 — Luciferase measurements reflect endogenous hepcidin mRNA expression. Expression fold-changes in the luciferase assay data (x axis) are strongly correlated with expression fold-changes in qPCR measurements of endogenous hepcidin mRNA (y axis). The green and blue data points indicate qPCR measurements for two independent biological replicates, each with technical replicates (n = 2). The data points correspond to the following stimulus concentrations: 6 ng/ml IL6, 25 ng/ml IL6, 200 ng/ml BMP6, 800 ng/ml BMP6, 200 ng/ml BMP6 + 25 ng/ml IL6, 800 ng/ml BMP6 + 25 ng/ml IL6, and 800 ng/ml BMP6 + 6 ng/ml IL6. The blue and green solid lines show linear fits to the data, and R indicates the Pearson correlation coefficient of each qPCR replicate series. See Supplemental Protocol S1 for a detailed description, and for the qPCR protocol. (JPG) [file pcbi.1003421.s001.jpg]

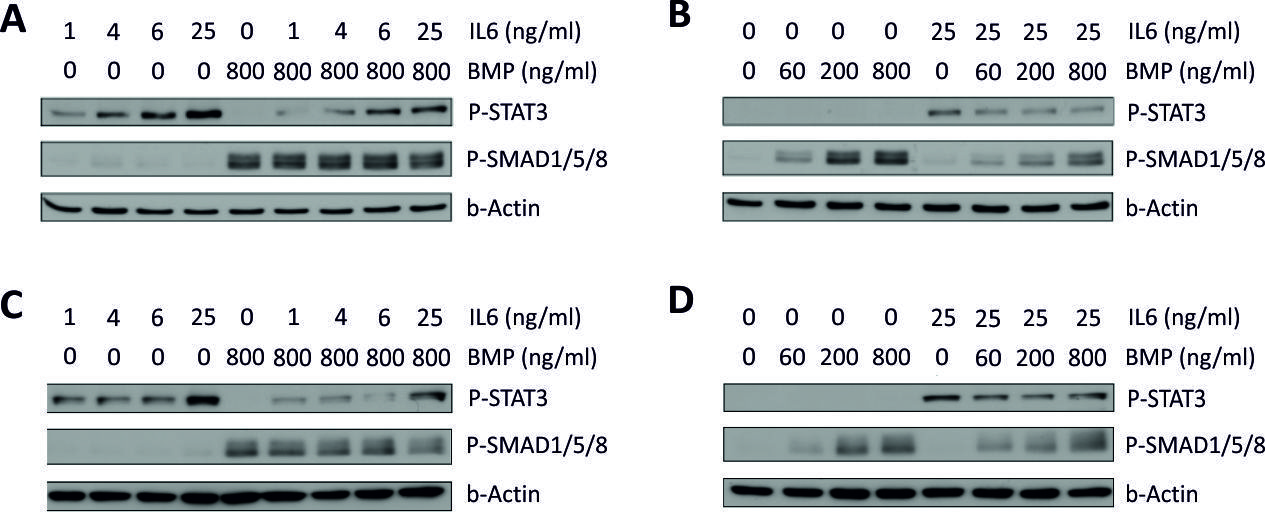

Supplement: Figure S2 — Immunoblotting of SMAD/STAT phosphorylation upon co-stimulation indicates moderate inhibitory signaling crosstalk. (A)–(D) HuH7 cells were stimulated with increasing doses of IL6 in the presence or absence of BMP (A, C) or vice versa (B, D). Signaling crosstalk was analyzed by immunoblotting against phosphorylated SMAD and STAT. Actin levels serve as loading controls. Two biological replicates were performed (Replicate 1: panels A and B; Replicate 2: panels C and D). (JPG) [file pcbi.1003421.s002.jpg]

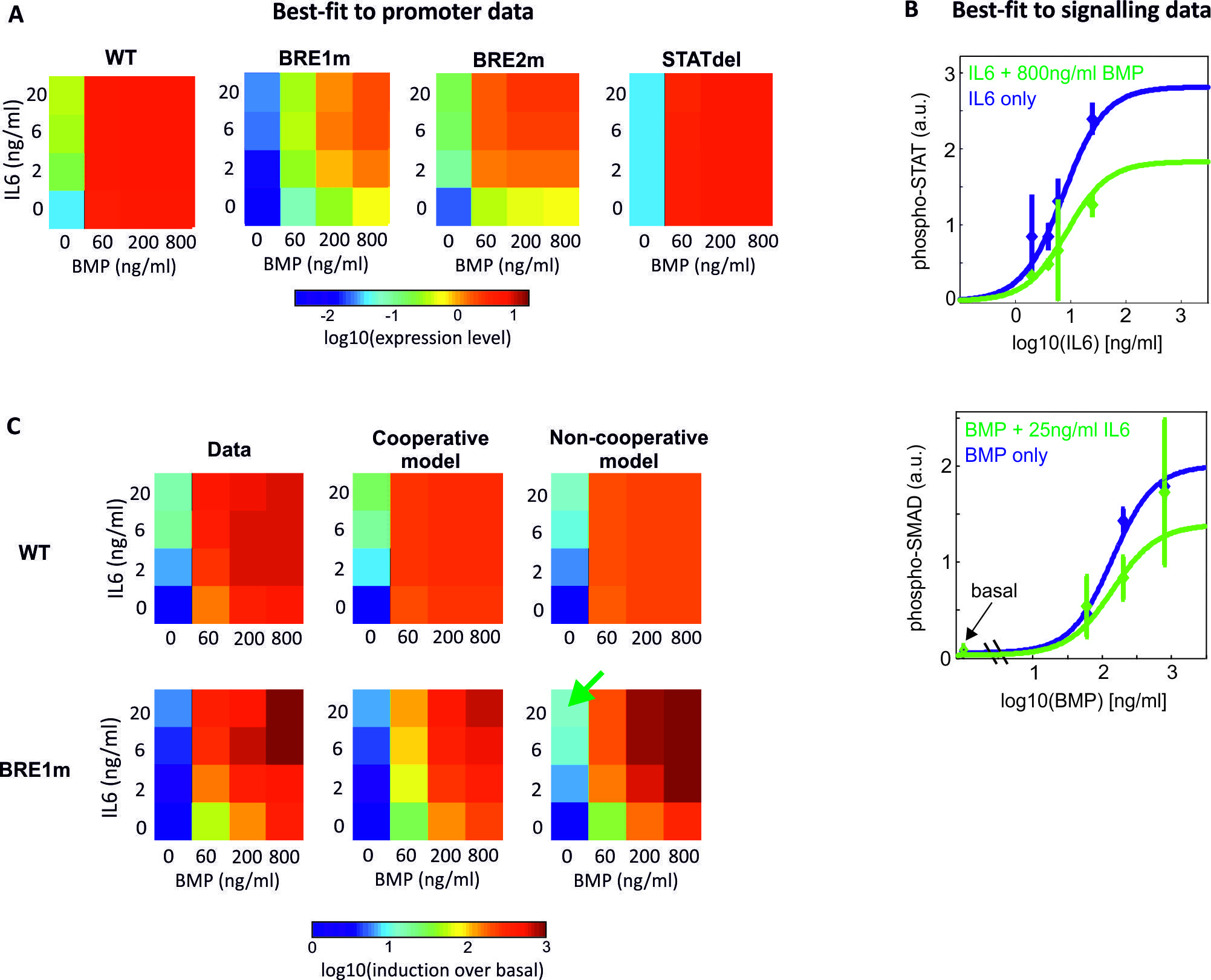

Supplement: Figure S3 — Fitting and analysis of a model with non-cooperative STAT and SMAD binding to STATBS and BRE1 sites. (A) and (B) Best-fit of the non-cooperative model (variant 1 in Fig. 2B) with inhibitory signaling crosstalk to luciferase data and dose-response curves of transcription factor phosphorylation (Supplemental Protocol S2). The simulated luciferase activities in A can be compared to the corresponding experimental data in Fig. 1C. Solid lines in B represent model trajectories in comparison to experimentally measured data points (shown as mean +/− std). (C) The non-cooperative model fails to explain the loss of IL6 sensitivity in the BRE1m promoter. Shown are the luciferase heatmaps of WT and BRE1m promoters (rows), as measured experimentally (left column) or simulated using cooperative and non-cooperative models, respectively (middle and right columns). Each heatmap was normalized to the corresponding basal expression level. The BRE1m promoter shows lower IL6 inducibility than WT in the data and in the cooperative model, but not in the non-cooperative model (indicated by green arrow). (JPG) [file pcbi.1003421.s003.jpg]

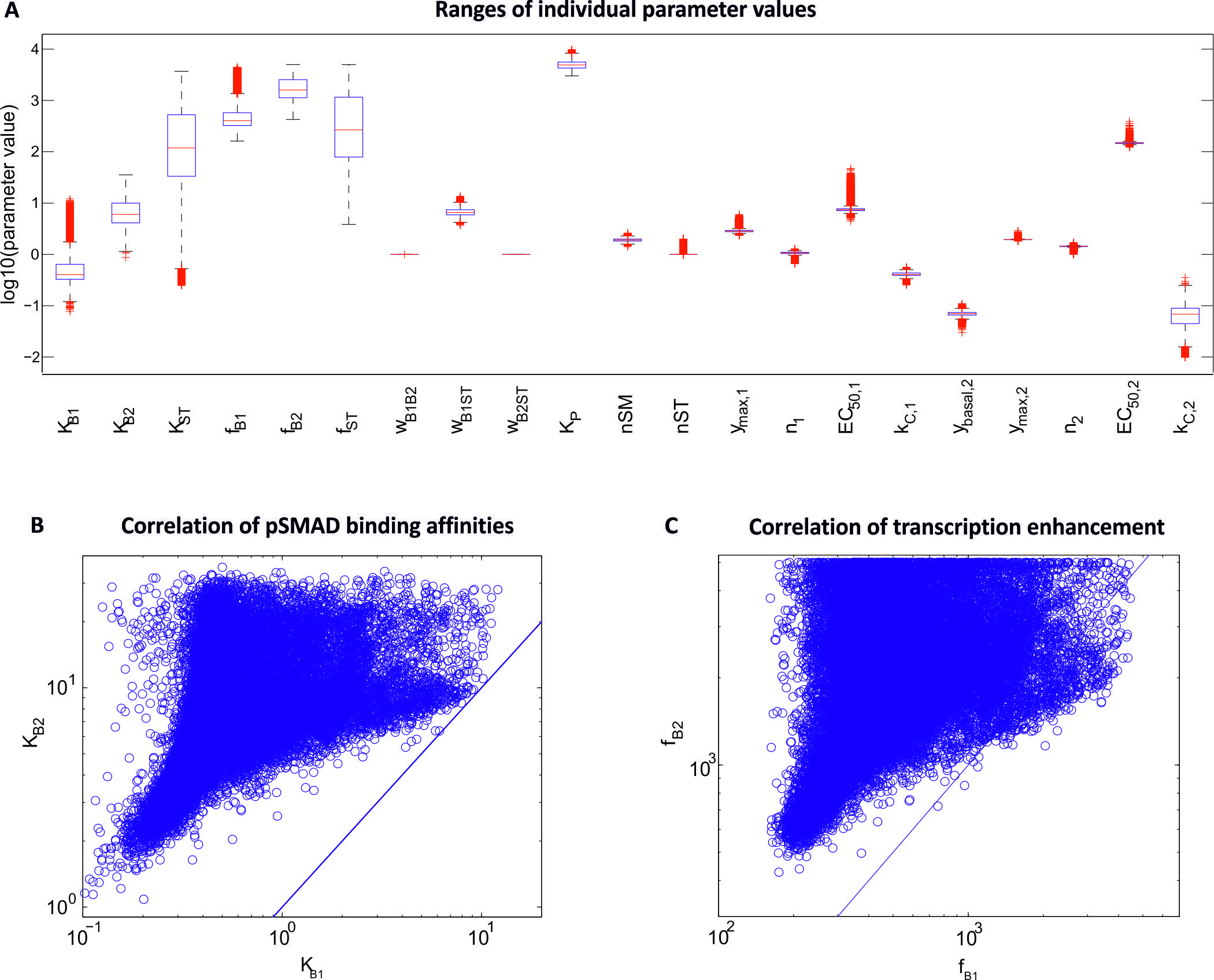

Supplement: Figure S4 — Analysis of parameter identifiability. (A) Box plots of the measurement-compliant parameter ranges. The model with inhibitory signaling crosstalk and BRE1-STATBS cooperativity (variant 4 in Fig. 2B) was analyzed, and parameter combinations with a similar goodness-of-fit (χ2<135) were collected (see Methods). The box plot indicate the distribution of each parameter (mid line: median; box edges: upper and lower quartile; whiskers contain 1.5 interquartile ranges from the edges; red crosses: outliers). (B) and (C) Relationship of model parameters describing the activities of BRE1 and BRE2. (B) Comparison pSMAD binding affinities of BRE1 and BRE2 (KB1 and KB2, respectively). (C) Comparison of RNAP interaction strength of BRE1-bound and BRE2-bound pSMAD (fB1 and fB2, respectively). Each circle corresponds to one measurement-compliant parameter (defined as in panel A), the solid line indicates the bisectrix. (JPG) [file pcbi.1003421.s004.jpg]

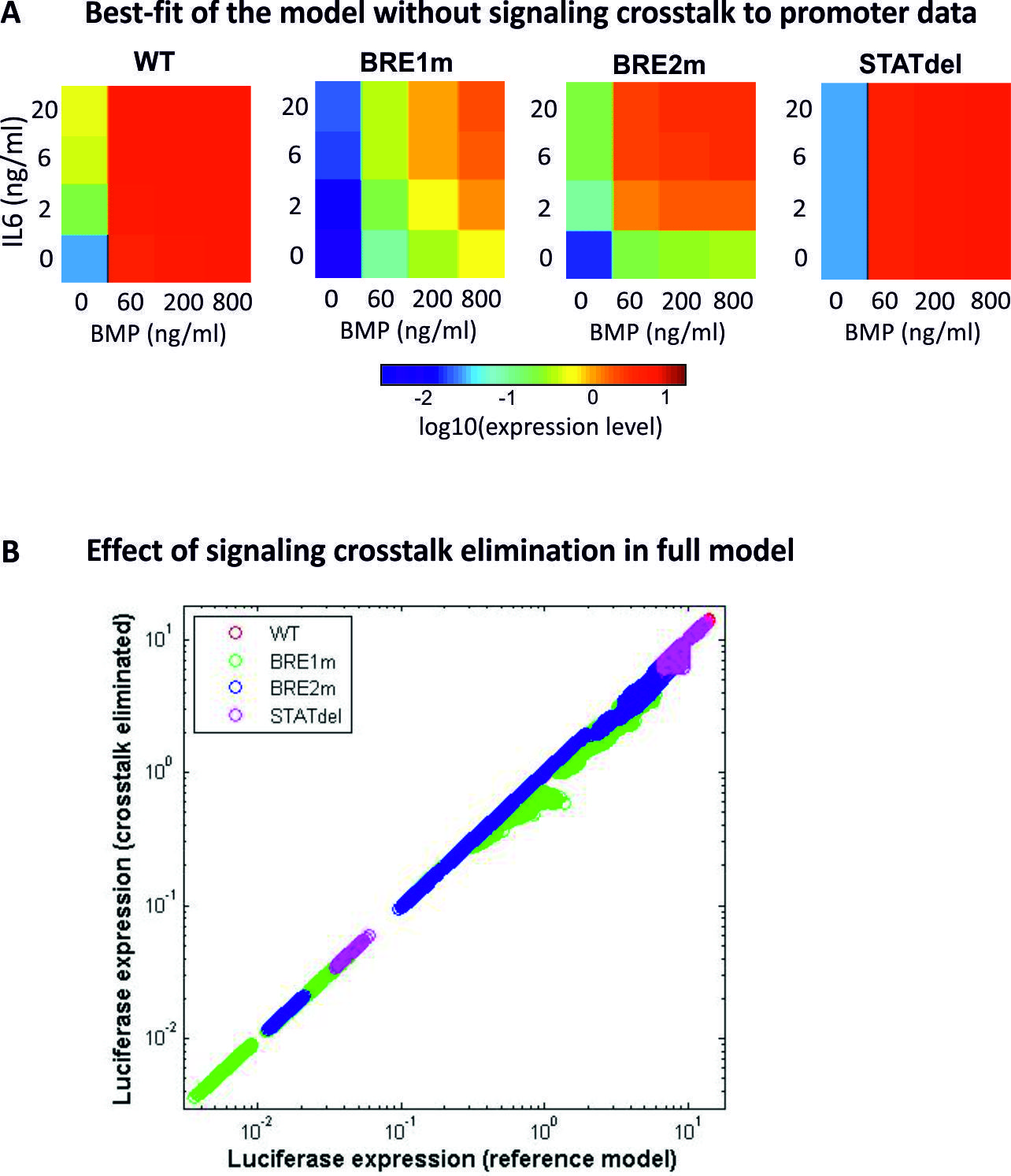

Supplement: Figure S5 — The luciferase measurements can be quantitatively modeled without assuming crosstalk between signaling pathways. (A) Best-fit of a hecipidin expression model without crosstalk at the level of BMP and IL6 signaling pathways. Luciferase expression was simulated using Eqs. S3.1 and S2.13 (Supplemental Text S3 and Supplemental Protocol S2), and the transcription rate in the model (pbound) was fitted to the data in Fig. 1C (using a scaling factor). The best-fit parameter values of this model are given in Supplemental Table S1. (B) Removal of signaling crosstalk does not appreciably affect the simulated luciferase activities in fits of the full model. The full model with signaling crosstalk was fitted to the data from multiple starting parameter sets, and all fitting solutions with a comparable goodness-of-fit (χ2<135) were analyzed (see Methods): The simulated luciferase levels with signaling crosstalk were plotted against the luciferase activities of model variants where crosstalk was eliminated (setting kC,1 and kC,2 in Eq. S3.1 to zero). The luciferase activities are essentially unaffected by the deletion of crosstalk. (JPG) [file pcbi.1003421.s005.jpg]

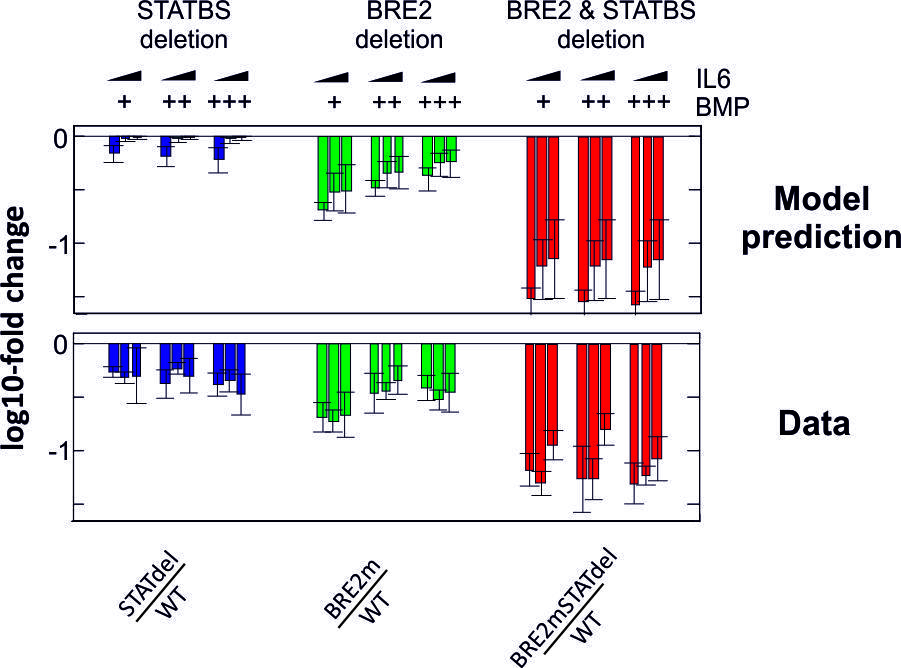

Supplement: Figure S6 — Buffering of BRE2 and STATBS single deletions. Systematic analysis of transcription factor binding site deletion effects confirms buffering of BRE2 and STATBS single deletions. The impact of binding site deletions was calculated by taking the luciferase activity ratios of different promoters (indicated at the bottom) and expressed as a log10-fold change (y axis). The fold-change upon a combined deletion of BRE2 and STATBS (red) is higher than the product of the single deletion fold-changes (green and blue) at high BMP stimulation. This indicates promoter saturation (see main text). Data points are mean and standard deviation, and model predictions represent the range of measurement-compliant parameter sets, as derived from a parameter identifiability analysis (see Methods). Co-stimulation conditions were considered to ensure that BRE2 and STATBS both contribute to expression. (JPG) [file pcbi.1003421.s006.jpg]

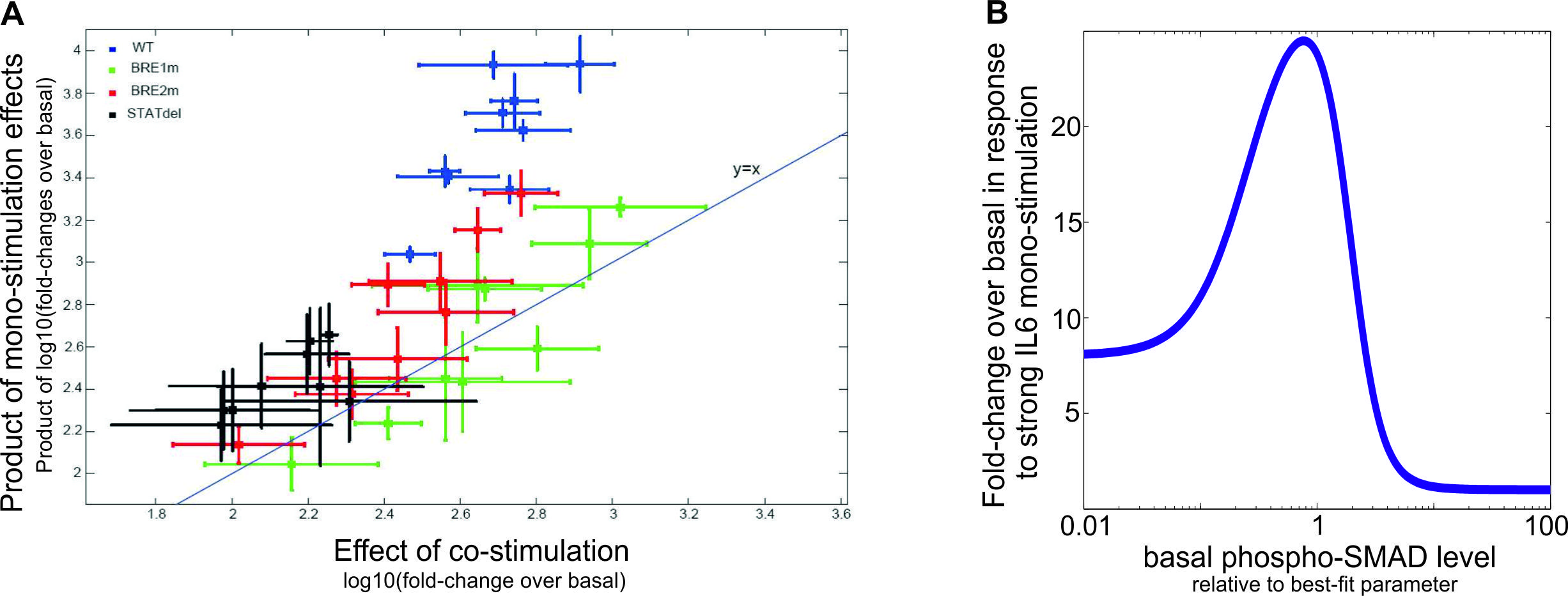

Supplement: Figure S7 — Integration of BMP and IL6 signals at the level of hepcidin expression. (A) The WT hepcidin promoter integrates BMP and IL6 signals in a sub-multiplicative manner, while mutants show multiplicative behavior. The x dimension shows the experimentally observed fold-expression-change over basal upon co-stimulation with BMP and IL6. The y dimension shows the product mono-stimulation responses over basal with the same doses of BMP and IL6, respectively. Each data point represents one co-stimulation condition (different concentrations of BMP and IL6 and/or different promoter constructs). The colors of the data points correspond to different promoter constructs (legend). The bisectrix (solid line) marks the expectation for a multiplicative system (Co-stimulation fold-change over basal equals the product of the mono-stimulation fold-changes). (B) Basal BMP signaling pathway activity is required for optimal IL6 responsiveness of the hepcidin promoter. The fold expression change in response to very strong IL6 mono-stimulation is shown as a function of the basal phospho-SMAD level (using the parameters of the best-fit WT model). Basal BMP signaling is required for optimal IL6 responsiveness of the promoter, indicating that both stimuli synergistically regulate hepcidin expression (i.e., in a more than multiplicative manner) in this regime of weak BMP signaling. This model prediction is supported by experiments in HuH7 cells showing that SMAD4 siRNA lowers the IL6 inducibility of the hepcidin promoter ([46]; unpublished observation), and with data in hepatocyte-specific SMAD4 knockout mice [52]. (JPG) [file pcbi.1003421.s007.jpg]
