# Supplementary material for: A Multi-Scale Model of Hepcidin Promoter Regulation Reveals Factors Controlling Systemic Iron Homeostasis
Source: PLoS Comput Biol. 2014 Jan 2;10(1):e1003421. doi: 10.1371/journal.pcbi.1003421 (PMC3879105; doi:10.1371/journal.pcbi.1003421)
Supplement: Protocol S1 — Comparison of luciferase signals with endogenous hepcidin mRNA expression. (PDF) [file pcbi.1003421.s009.pdf]

Supplemental Protocol S1 – Comparison of luciferase signals with endogenous hepcidin mRNA expression

Luciferase reporter assays allowed measuring the activity of WT and mutant hepcidin promoter versions under various experimental conditions. QPCR measurements of endogenous hepcidin mRNA were performed under the same experimental conditions to confirm that the reporter measurements reflect endogenous hepcidin expression. In our previous work, we systematically compared qPCR and luciferase signals (fold-change over basal expression) over various stimulation and knockdown conditions ([1]; unpublished observations). We found a high correlation between both experimental methods which confirms that luciferase measurements faithfully reflect endogenous regulation. Moreover, this supports our model assumption that post-transcriptional regulation of hepcidin expression is negligible.

In this study, we performed luciferase assays for higher doses of IL6 and BMP than in the previous work. It was therefore necessary to conduct additional qPCR measurements to confirm that luciferase activities reflect endogenous expression in this concentration regime as well. The results of this analysis at high BMP and IL6 concentrations are shown Supplemental Fig. S1. In the following, we will briefly describe the experimental procedure that was used to gather endogenous expression by qPCR: HUH7 cells (1.5 X 10^5 per well) were seeded onto 6-well plates and the day after the culture medium was exchanged to fetal calf serum-free medium. After 12 hours the cells were treated with increasing doses of BMP-6 (60; 200; 800 ng/mL; R&D Systems) and/or IL-6 (2; 4; 6; 25 ng/mL; R&D Systems) for 12 hours and then harvested for extraction of total RNA. Total RNA was isolated using the Qiagen RNAeasy kit according to the manufacturer's instruction. Total RNA (1 µg) was reverse-transcribed in a 25 µl reaction mixture using MMLV (Moloney-murine-leukaemia virus) reverse transcriptase (Fermentas) and random oligomers as primers. SYBR green quantitative real-time PCR (qRT-PCR) was performed using the ABI StepONE Plus realtime PCR system (Applied Biosystems) with the following primers: hs_GAPDH-F, CATGAGAAGTATGACAACAGCCT; hs_GAPDH-R, AGTCCTTCCACGATACCAAAGT; hs_HAMP-F, CTCTGTTTTCCCACAACAGAC; and hs_HAMP-R, TAGGGGAAGTGGGTGTCTC. Relative hepcidin mRNA expression was normalized to GAPDH (glyceraldehyde-3-phosphate dehydrogenase) mRNA. Results were calculated using the Pfaffl method [2].

1. Mleczko-Sanecka K, Casanovas G, Ragab A, Breitkopf K, Muller A, et al. (2010) SMAD7 controls iron metabolism as a potent inhibitor of hepcidin expression. Blood 115: 2657-2665.
2. Pfaffl MW (2001) A new mathematical model for relative quantification in real-time RT-PCR. Nucleic acids research 29: e45.
