# Supplementary material for: A Multi-Scale Model of Hepcidin Promoter Regulation Reveals Factors Controlling Systemic Iron Homeostasis
Source: PLoS Comput Biol. 2014 Jan 2;10(1):e1003421. doi: 10.1371/journal.pcbi.1003421 (PMC3879105; doi:10.1371/journal.pcbi.1003421)
Supplement: Protocol S2 — Model implementation, model fitting and model selection. (PDF) [file pcbi.1003421.s010.pdf]

Supplemental Protocol S2 – Model implementation, model fitting and model selection

During our modeling analyses, we fitted multiple variants of the model to the experimental data. In the following, we will describe the implementation of different model variants before discussing the model fitting strategy and the model selection procedure.

Implementation of model variants: We analyzed eight variants of the promoter model (Fig. 2B). All model variants contained well-known aspects of promoter regulation such as pSTAT/pSMAD binding to the promoter and RNAP activation by transcription factors (grey arrows in Fig. 2B). In the full model (topology 8 in Fig. 2B), we additionally allowed all possible cooperative protein-protein interactions between transcription factors (indicated by red arrows in Fig. 2B). This full model is described by the thermodynamic promoter model derived in Supplemental Text S2 (Eq. S2.10). In the context of hepcidin promoter regulation, Eq S2.10 may be rewritten as (S2.13)

$$p_{bound} = \frac{F_{Reg}}{F_{Reg}+K_P}$$

$$F_{Reg} = \frac{1+\frac{[pSM]^{nSM}}{K_{B1}}f_{B1}+\frac{[pSM]^{nSM}}{K_{B2}}f_{B2}+\frac{[pST]^{nST}}{K_{ST}}f_{ST}+\frac{[pSM]^{2nSM}}{K_{B1}K_{B2}}f_{B1}f_{B2}\omega_{B1B2}+\frac{[pSM]^{nSM}[pST]^{nST}}{K_{B1}K_{ST}}f_{B1}f_{ST}\omega_{B1ST}+\frac{[pSM]^{n_{SM}}[pST]^{nST}}{K_{B2}K_{ST}}f_{B2}f_{ST}\omega_{B2ST}+\frac{[pSM]^{nSM}[pST]^{nST}}{K_{B1}K_{B2}K_{ST}}f_{B1}f_{B2}f_{ST}\omega_{B1B2}\omega_{B1ST}\omega_{B2ST}}{1+\frac{[pSM]^{nSM}}{K_{B1}}+\frac{[pSM]^{nSM}}{K_{B2}}+\frac{[pST]^{nST}}{K_{ST}}+\frac{[pSM]^{2nSM}}{K_{B1}K_{B2}}\omega_{B1B2}+\frac{[pSM]^{nSM}[pST]^{nST}}{K_{B1}K_{ST}}\omega_{B1ST}+\frac{[pSM]^{n_{SM}}[pST]^{nST}}{K_{B2}K_{ST}}\omega_{B2ST}+\frac{[pSM]^{nSM}[pST]^{nST}}{K_{B1}K_{B2}K_{ST}}\omega_{B1B2}\omega_{B1ST}\omega_{B2ST}}$$

Here, we have replaced the generic transcription factor concentrations A_i by the levels of phosphorylated SMAD and STAT transcription factors, designated as [pSM] and [pST], respectively.

Transcription factors of the SMAD and STAT families homo- and hetero-oligomerize upon phosphorylation, and oligomerization is thought to be important for gene expression regulation [1,2]. Oligomerization would result in a nonlinear relationship between the level of transcription factors and gene expression. To accommodate possible oligomerization reactions we allowed that pSM and pST control hepcidin expression with the exponents n_{SM} and n_{ST}, respectively. The values of n_{SM} and n_{ST} were allowed to vary between 1 and 3 during fitting to reflect dimerization and trimerization reactions. An exponent larger than one for SMAD signaling improved the fitting result, while $n_{ST} > 1$ was not beneficial (see Supplemental Table S1 for best-fit parameters and not shown).

The affinity of the transcription factors for the BRE1, the BRE2 and the STATBS is described by the corresponding dissociation constants (K_{B1}, K_{B2}, K_{ST}). Each transcription factor bound to DNA contacts polymerase, and enhances RNAP recruitment to the TSS with the binding energies f_{B1}, f_{B2} and f_{ST} (denoting the effects of transcription factors bound to BRE1, BRE2 and STATBS, respectively). The half-maximal saturation parameter K_P takes into account that the TSS may be fully occupied by RNAP, implying that a further increase in gene expression is not possible if $F_{Reg} >> K_P$. Protein-protein interactions among transcription factors on DNA may mutually enhance promoter binding and/or RNAP activation; in the model, such

complex formation is described by the binding energies ω_{B1B2} (bridging between transcription factors bound to BRE1 and BRE2), ω_{B1ST} (bridging between BRE1 and STATBS) and ω_{B2ST} (bridging between BRE2 and STATBS).

We also analyzed the ability of simpler model variants (topology 1-7 in Fig. 2B) to fit the experimental data which allowed us to derive a minimal essential model of hepcidin promoter regulation. These simpler variants contained only a subset of all possible cooperative protein-protein interactions between transcription factors (red arrows in Fig. 2B). Cooperative interactions were eliminated by fixing the parameters ω_{ij} of the forbidden cooperative protein-protein interaction to unity (Eq. S2.13). For example, the minimal model variant 1 did not contain any of the hypothetical protein-protein-interactions among transcription factors (i.e., the corresponding interaction parameters in Eq. S2.13 were set to $\omega_{B1B2} = \omega_{B1ST} = \omega_{B2ST} = 1$).

Model fitting to the luciferase data in Fig. 1C required the simulation of promoter mutants. Equation S2.13 describes the wildtype construct, but was adjusted to mutant promoters by eliminating transcription factor binding terms (i.e., by setting $[pSM]/K_{B1}$, $[pSM]/K_{B2}$ and/or $[pST]/K_{ST}$ to zero).

Taken together, we use relatively simple equations (Eqs. S3.1-S3.3 and S2.13) to simultaneously describe steady state crosstalk at the signaling and promoter levels. The promoter model (Eq. S2.13; 10 parameters) was either combined with a signaling model that does not take into account pathway crosstalk (Eq. S3.1; 7 parameters; best-fit in Supplemental Fig. S5) or with a signaling model considering signaling crosstalk (Eq. S3.2 and S3.3; 9 parameters; best-fit in Fig. 2C/D). During model fitting, the model species [pSM] and [pST] (Eqs. S3.1-S3.3) were assumed to reflect the degree of transcription factor phosphorylation, while p_{bound} (Eq. S2.12) scales with luciferase expression levels.

Fitting strategy: All model variants were fitted to the data by minimizing the χ^2 metric ($\chi^2 = (M_i - s_j \cdot D_i)^2/\sigma_i^2$; where M_i, D_i and σ_i are the simulated value, the measured value and the experimental error, respectively). The model species [pSM] and [pST] were fitted to the transcription factor phosphorylation data, while p_{bound} was fitted to the luciferase measurements. A scaling factor s_j was used to adjust the simulated transcription rate (p_{bound}; Eq, S3.13) to the luciferase data. Transcription factor phosphorylation was formulated in arbitrary units (Eqs. S3.1-S3.3; Supplemental Table S1), and could thus be fitted without the use of a scaling factor (by adjusting $y_{max,1}$ and $y_{max,2}$). All parameters of the model were allowed to vary within a physiologically reasonable range, and the ranges as well as the best-fit values are given in Table S1. Parameter optimization was done using a deterministic trust region optimizer in Matlab. In order to circumvent local minima, we repeatedly fitted the model starting from 80.000 quasi randomly distributed positions in the space of allowed parameter ranges. The results of this repeated fitting strategy apparently converged to a global optimum, since ~50% of the fitting runs yielded χ values close to the minimum all 80.000 runs.

Model selection: To derive a minimal essential model of hepcidin regulation, we employed a model selection approach considering model topologies of different complexity, and systematically compared their ability to fit the experimental observations (Fig. 2B). Models of different complexities were compared based on their goodness-of-fit to the training data using the Akaike Information criterion and the Likelihood ratio test. These statistical criteria indicate whether a more complex model with more parameters fits the data significantly better than simpler variants. Briefly, we used the following formula for the Akaike information criterion (S2.14)

$$AIC = \chi^2 + 2 \cdot k$$

Here, k is the number of parameters of that model. The model with the least AIC was taken as the variant that is most suited to describe the data. The likelihood ratio statistic (S2.15)

$$D = {\chi_2}^2 - {\chi_1}^2$$

equals the difference in the χ^2 values of two nested models. Nested means that the larger model (characterized by ${\chi_2}^2$) must contain the smaller model (characterized by ${\chi_1}^2$), and can thus be interconverted into the smaller variant by appropriate parameter choice. D was compared to a tabulated value of the χ^2 statistic using the difference in the number of model parameters as the degree of freedom. If the tabulated values were less than D we rejected H0,null hypothesis that the extra parameters are necessary for a better model

Both statistical measures revealed that a relatively simple model containing a single protein-protein interaction among SMAD and STAT transcription factors bound to BRE1 and the nearby STATBS was sufficient to explain the data (model 4 in Fig. 2B). The best fitting result of the BRE1-STATBS interaction model with signaling crosstalk is shown in Figs. 2C and D. Statistical measures indicate that the model matched the data close to experimental measurement noise (χ^2 = 124, N = 80).

Parameter identifiability analysis: Parameter identifiability was analysed using the strategy proposed by Hengl et al. [3]: Briefly, the parameter vectors of the top 45% fitting results had a similar goodness of fit (χ^2 < 135), and were analysed with respect to parameter ranges and parameter correlations. The robustness of model predictions was estimated by repeatedly simulating predictions for the top 45% of the model solutions. The upper and lower bounds of the computed solution are given as a prediction range in Fig. 3.

1. Horvath CM (2000) STAT proteins and transcriptional responses to extracellular signals. Trends in biochemical sciences 25: 496-502.
2. Massague J, Seoane J, Wotton D (2005) Smad transcription factors. Genes & development 19: 2783-2810.
3. Hengl S, Kreutz C, Timmer J, Maiwald T (2007) Data-based identifiability analysis of non-linear dynamical models. Bioinformatics 23: 2612-2618.
