# Supplementary material for: A Multi-Scale Model of Hepcidin Promoter Regulation Reveals Factors Controlling Systemic Iron Homeostasis
Source: PLoS Comput Biol. 2014 Jan 2;10(1):e1003421. doi: 10.1371/journal.pcbi.1003421 (PMC3879105; doi:10.1371/journal.pcbi.1003421)
Supplement: Table S1 — Best-fit model parameters. Best-fit parameters for the models with and without signaling crosstalk (Figs. 2C/D and Supplemental Fig. S5, respectively). The parameters 1–9 belong to the signaling module of the model, while the remaining ones describe promoter regulation. Each parameter was constrained to a physiologically feasible range during fitting. The Hill coefficients of the signaling module (parameters 3 and 7) were restricted to values typical for biochemical response curves. The other parameters of the signaling module (ymax, EC50) represent a combination of multiple signaling reaction constants, and were thus constrained such that they match the experimental measurements of transcription factor phosphorylation (Fig. 1D). Most parameter ranges of the promoter module were taken from the literature. Some were allowed to vary over a broad range to accommodate different kinds of qualitative behavior. For example, the wide range of half-maximal promoter saturation constants (KP; parameter 10) allows for promoter saturation to occur upon stimulation. Likewise, the KD values of transcription factor binding to cognate promoter sites (parameters 11–13) were allowed to vary over a broad range to accommodate weak and strong binding. The parameter ranges for the constants describing protein-protein interactions on the promoter (parameters 14–19) represent the typical interaction energies of 1–5 kcal/mol reported in the literature [29]. The exponents nSM and nST reflect transcription factor dimerization and trimerization, implying that values of up to 3 can be expected. (PDF) [file pcbi.1003421.s011.pdf]

Supplemental Table S1 – Best-fit model parameters

#	Parameter	Description	Fitting range	Value with signaling crosstalk (Fig. 2C and D)	Value without signaling crosstalk (Fig. S5)
1	$y_{max,1}$	Maximal STAT activation	0.5 – 7 (a.u.)	2.8520	0.2598
2	$EC_{50,1}$	Half-maximal IL6 concentration for STAT activation	0.5 – 50 (ng/ml)	7.7388	2.5048
3	n_1	Hill coefficient STAT activation	0.5 - 5	1.0242	2.3717
4	$Y_{basal,2}$	Basal SMAD activation	0.03 – 0.11 (a.u.)	0.0583	0.0458
5	$y_{max,2}$	Maximal SMAD activation	1.5 – 3 (a.u.)	1.9490	33.3224
6	$EC_{50,2}$	Half-maximal BMP concentration for SMAD activation	20 – 5000 (ng/ml)	140.2440	464.7168
7	n_2	Hill coefficient SMAD activation	1 - 5	1.4481	1.2969
8	$k_{C,1}$	Cross-inhibition of STAT signaling by pSMAD	0.01 - 5	0.4135	-
9	$k_{C,2}$	Cross-inhibition of SMAD signaling by pSTAT	0.01 - 5	0.1285	-
10	K_P	Half-maximal promoter saturation	1 - 10000	6804.7	5413.9
11	K_{B1}	K_D of pSMAD binding to BRE1	0.001 - 5000	0.4391	1.6370
12	K_{B2}	K_D of pSMAD binding to BRE2	0.001 - 5000	16.8738	94.8517
13	K_{ST}	K_D of pSTAT binding to STATBS	0.001 - 5000	206.3988	339.9842
14	f_{B1}	Binding of BRE1-bound pSMAD to RNAP	1 - 5000	537.6490	257.3671
15	f_{B2}	Binding of BRE2-bound pSMAD to RNAP	1 - 5000	4972.6	2777.9
16	f_{ST}	Binding of STATBS-bound pSTAT to RNAP	1 - 5000	584.75	4691.8
17	ω_{B1B2}	cooperativity between BRE1 and BRE2	1 - 300	1 (fixed)	1 (fixed)

18	ω_{B1ST}	cooperativity between BRE1 and STATBS	1 - 300	5.3869	6.1287
19	ω_{B2ST}	cooperativity between BRE2 and STATBS	1 - 300	1 (fixed)	1 (fixed)
20	n_{SM}	Exponent reflecting pSMAD oligomerization	1 - 3	1.7807	1 (fixed)
21	n_{ST}	Exponent reflecting pSMAD oligomerization	1 - 3	1	1 (fixed)
