# Supplementary material for: A Multi-Scale Model of Hepcidin Promoter Regulation Reveals Factors Controlling Systemic Iron Homeostasis
Source: PLoS Comput Biol. 2014 Jan 2;10(1):e1003421. doi: 10.1371/journal.pcbi.1003421 (PMC3879105; doi:10.1371/journal.pcbi.1003421)
Supplement: Text S1 — Conceptual model of systemic iron homeostasis. (PDF) [file pcbi.1003421.s012.pdf]

Supplemental Text S1 – Conceptual model of systemic iron homeostasis

Systemic iron homeostasis is maintained by an auto-regulatory negative feedback loop. Iron overload triggers hepcidin expression, and hepcidin in turn lowers intestinal iron influx (Fig. 4D). Such feedback regulation is thought to compensate for fluctuations in dietary iron content [1]. We analyzed a minimal mathematical model of this circuitry to gain insights into iron homeostasis and the factors determining iron blood levels.

The dynamics of the model species were described using the framework of ordinary differential equations. The model equations read (S1.1)

$$\frac{d[Fe_b]}{dt} = v_{influx} - v_{efflux} = \frac{k_{influx}}{1+k_{FB}\cdot[hepcidin]}\cdot[Fe_i] - k_{efflux}\cdot[Fe_b]$$

$$\frac{d[hepcidin]}{dt} = v_{synthesis} - v_{degradation} = f([Fe_b]) - k_{deg}\cdot[hepcidin]$$

Iron blood levels were described by the species Fe_b, whose levels are controlled by influx and efflux reactions. The iron influx rate is proportional to the intestinal iron concentration (species Fe_i). The efflux rate is modeled as a first-order process, and represents iron excretion and incorporation into red blood cells. Iron blood levels are assumed to control hepcidin expression via the BMP signaling pathway, and this is described by the function $f([Fe_b])$. Hepcidin levels are additionally controlled by first-order degradation (k_{deg}) following its uptake into ferroportin-expressing cells [1]. Negative feedback regulation was considered in the model by assuming that the iron influx is negatively influenced by hepcidin. The feedback strength is determined by the parameter k_{FB}.

For the analysis of long-term iron homeostasis, we are not interested in the temporal dynamics of the system and focus on the steady state behavior ($d[Fe_b]/dt = 0$ and $d[hepcidin]/dt = 0$). Thus, Eq. S1.1 simplifies to a set of algebraic equations that can be solved for the steady state concentration of iron in the blood.

In the most general form, the synthesis function at the steady state of interest is $f([Fe_b]) = \mathrm{k_{induced}} * [Fe_b]^R$. The parameter $k_{induced}$ is a constant that is independent of the iron blood concentration. R is the gain of the synthesis rate with respect to the iron blood concentration at the steady state of interest, i.e., $R = d\ln(v_{synthesis})/d\ln(Fe_b)$. The gain is a normalized slope that describes how a percent change in the iron blood concentration translates into a percent change in the hepcidin synthesis rate. The hepcidin synthesis rate depends in a linear fashion on the iron blood concentration for R = 1. A gain of R > 1 implies cooperative regulation, while R = 0 implies that hepcidin synthesis is not regulated by the iron blood concentration. R thus reflects the steepness of the hepcidin promoter dose-response curve with respect to iron/BMP stimulation.

In the limit of strong feedback (large k_{FB}) we can approximate the steady state as (S1.2)

$$[Fe_b] = \left(\frac{k_{influx} \cdot [Fe_i] \cdot k_{deg}}{k_{efflux} \cdot k_{induced} \cdot k_{FB}}\right)^{1/(1+R)}$$

This equation implies that the steady state iron blood level responds to a change in iron diet content $[Fe_i]$ in a less than linear manner as long as $R > 0$, since $[Fe_i]$ enters as the (R+1)-th root only. We conclude that our simple model reflects partial homeostasis. Homeostasis is especially pronounced if hepcidin expression responds in a steep and nonlinear manner to changes in the iron blood level ($R > 1$). A loss of homeostasis is observed for $R = 0$, i.e., if the hepcidin promoter shows basal or strongly saturated expression, and therefore does not respond to changes in the iron blood level. The range of $[Fe_i]$ concentrations where homeostasis is observed is therefore determined by the dynamic range of the hepcidin promoter response. We confirmed by numerical simulations that similar principles hold for the extended homeostasis model (discussed in Supplemental Text S4 and shown in Figs. 4D and E).

Taken together the steepness of the hepcidin promoter response towards iron-BMP signaling is the key parameter controlling how well the homeostasis loop compensates fluctuations in diet iron levels (in terms of the fold-changes in $[Fe_b]$ induced by a certain fold-change in $[Fe_i]$). The other model parameters (k_{influx}, k_{deg}, k_{efflux}, $k_{induced}$, k_{FB}) determine the absolute iron blood concentration, but do not affect the degree of homeostasis in the model.

An important aspect of iron homeostasis is the transcriptional regulation of hepcidin expression by iron-independent stimuli such as IL6. Strong hepcidin regulation by IL6 reduces the gain R of the hepcidin synthesis rate with respect to iron blood levels, and thereby diminishes homeostasis as shown in the following. Consider the following extended model which takes into account that hepcidin synthesis occurs by an iron-independent term (k_{basal}) (S1.3)

$$\frac{d[Fe_b]}{dt} = v_{influx} - v_{efflux} = \frac{k_{influx}}{1+k_{FB} \cdot [hepcidin]} \cdot [Fe_i] - k_{efflux} \cdot [Fe_b]$$

$$\frac{d[hepcidin]}{dt} = v_{synthesis} - v_{degradation} = k_{basal} + k_{induced} * [Fe_b] - k_{deg} \cdot [hepcidin]$$

Here, we assumed that hepcidin expression by iron occurs with moderate steepness ($n = 1$). The following steady state equation can be derived for the limit of strong feedback (i.e., large k_{FB}) (S1.4)

$$[Fe_b] = \frac{1}{2} \cdot \left(-\frac{k_{basal}}{k_{induced}} + \sqrt{\left(\frac{k_{basal}}{k_{induced}}\right)^2 + 4 \cdot \frac{k_{influx} \cdot [Fe_i] \cdot k_{deg}}{k_{efflux} \cdot k_{induced} \cdot k_{FB}}}\right)$$

This equation reveals that the intestinal iron concentration ($[Fe_i]$) enters in a square-root manner for $k_{basal} = 0$, confirming that the system without basal expression shows partial

homeostasis. For non-zero basal expression the iron blood concentration can depend on the intestinal iron concentration in a linear manner, i.e., homeostasis may be lost. This emergence of linear behavior is not immediately obvious from Eq. S1.4. Linear behavior can be shown by calculating the gain of the iron blood concentration with respect to the intestinal iron level (S1.5).

$$G = \frac{[Fe_i]}{[Fe_b]} \cdot \frac{d[Fe_b]}{d[Fe_i]}$$

Using l'Hopitals' rule it can be shown that

$$\lim_{k_{basal}/k_{induced} \to \infty}(G) = 1$$

Thus, a linear relationship between intestinal and blood iron levels, i.e., a loss of homeostasis, is observed for sufficiently high iron-independent, basal hepcidin expression. We conclude that the regulation of hepcidin by other signaling pathways other than BMP may modulate how strongly iron diet content fluctuations can be compensated.

Whether iron homeostasis is compromised by iron-independent regulators of hepcidin expression, however, depends on the mode of signal integration at the hepcidin promoter: In Eq. S1.3, it was assumed that the iron-BMP-signaling axis and the iron-independent terms control hepcidin expression in an additive manner (logical OR gate). Alternatively, two pathways may control hepcidin expression multiplicatively (logical AND gate). This scenario is covered by the differential equation system Eq. S1.1: In this model, the iron-independent pathway would change the value of the synthesis rate constant $k_{induced}$. From Eq. S1.2, we learned that the degree of homeostasis in this system is only determined by the steepness of promoter regulation by BMP, but not by other parameters such as $k_{induced}$. We conclude that iron-independent pathways would *not* modulate how strongly iron diet content fluctuations can be compensated in the multiplicative scenario. Instead, the iron-independent pathways would modulate the absolute iron blood levels, although to a limited extent only.

We conclude that the performance of the iron homeostasis loop is determined by the steepness of the promoter response towards BMP stimulation. Whether or not this steepness (and thus iron homeostasis) is modulated by iron-independent regulators of hepcidin expression depends on the mode of signal integration at the hepcidin promoter. We therefore set out to systematically quantify hepcidin promoter regulation by BMP and IL6 using systematic promoter mutagenesis and co-stimulation experiments.

1. Hentze MW, Muckenthaler MU, Galy B, Camaschella C (2010) Two to tango: regulation of Mammalian iron metabolism. Cell 142: 24-38.
