# Supplementary material for: A Multi-Scale Model of Hepcidin Promoter Regulation Reveals Factors Controlling Systemic Iron Homeostasis
Source: PLoS Comput Biol. 2014 Jan 2;10(1):e1003421. doi: 10.1371/journal.pcbi.1003421 (PMC3879105; doi:10.1371/journal.pcbi.1003421)
Supplement: Text S2 — Derivation of a thermodynamic model for the hepcidin promoter. (PDF) [file pcbi.1003421.s013.pdf]

Supplemental Text S2 – Derivation of a thermodynamic model for the hepcidin promoter

The hepcidin promoter consists of three major regulatory elements, all of which are controlled by IL6 and/or BMP signaling [1]: (i) the STAT-binding site (STATBS); (ii) and (iii) the BMP-responsive elements 1 and 2 (BRE1 and 2). When occupied by cognate transcription factors, each of these sites may directly recruit RNA polymerase II and thereby initiate transcription. Additionally, the transcription factors may mutually enhance their impact on transcription, e.g., by cooperative promoter binding, DNA looping or opening of chromatin.

A thermodynamic model of transcription was derived in order to quantitatively describe signal integration by the hepcidin promoter (reviewed in [2-4]). Thermodynamic modeling applies methods from statistical thermodynamics to describe combinatorial binding of transcription factors to promoters. The approach additionally takes into account protein-protein interactions on the promoter: (i) transcription factors may contact RNAP, and thereby promote RNAP recruitment and transcription. (ii) transcription factors may form pairwise complexes, thus cooperatively enhancing their promoter binding or transcriptional activation. In the following, we will derive a thermodynamic model comprising all possible pairwise protein-protein interactions on a promoter containing three transcription factor binding sites. As described in Supplemental Protocol S3, we reduced the complexity of this model based on model fitting and model selection approaches.

A central concept in thermodynamic modeling are the so-called promoter states which represent the transcription factor binding configurations of a promoter. Combinatorial binding of transcription factors to three specific binding sites in the promoter and polymerase binding to the transcription start site (TSS) gives rise to 2^4 = 16 promoter states (Fig. 2A).

Thermodynamic modeling assumes that the transcription initiation rate is proportional to the amount of polymerase bound to the promoter. We therefore derive an expression for the probability of polymerase binding based on the promoter states. The probability of polymerase binding is given by the sum over the statistical weights of the polymerase-bound promoter states divided by the sum over all promoter weights (S2.1)

$$p_{bound} = \frac{\sum Z_{bound}}{Z_{tot}}$$

We use the notation ‘P’ to describe empty promoter states where all P polymerase molecules are bound non-specifically to genomic DNA (or diffuse freely in the nucleoplasm). ‘P-1’ refers to the active promoter scenario where one polymerase molecule is bound specifically to the TSS, and the remaining P-1 molecules show background or no binding.

Using a similar nomenclature for each of the activating transcription factor binding sites A_i, we can write the sum over the statistical weights of the polymerase-bound states as (S2.2)

$$\sum Z_{bound} = Z(P-1, A_1, A_2, A_3) + Z(P-1, A_1-1, A_2, A_3) + Z(P-1, A_1, A_2-1, A_3) + Z(P-1, A_1, A_2, A_3-1) + Z(P-1, A_1-1, A_2-1, A_3) + Z(P-1, A_1-1, A_2, A_3-1) + Z(P-1, A_1, A_2-1, A_3-1) + Z(P-1, A_1-1, A_2-1, A_3-1)$$

The total statistical weight additionally takes into account 8 polymerase-free promoter states (S2.3)

$$Z_{tot} = \sum Z_{bound} + \sum Z_{free} = \sum Z_{bound} + Z(P, A_1, A_2, A_3) + Z(P, A_1-1, A_2, A_3) + Z(P, A_1, A_2-1, A_3) + Z(P, A_1, A_2, A_3-1) + Z(P, A_1-1, A_2-1, A_3) + Z(P, A_1-1, A_2, A_3-1) + Z(P, A_1, A_2-1, A_3-1) + Z(P, A_1-1, A_2-1, A_3-1)$$

The weights of the 16 individual promoter states are given by (S2.4)

$$Z(P, A_1, A_2, A_3) = \frac{N_{NS}! * e^{-\frac{\varepsilon_P^{NS}+\varepsilon_{A_1}^{NS}+\varepsilon_{A_2}^{NS}+\varepsilon_{A_3}^{NS}}{k_B*T}}}{P! * A_1! * A_2! * A_3! * (N_{NS}-P-A_1-A_2-A_3)!}$$

$$Z(P, A_1-1, A_2, A_3) = \frac{N_{NS}! * e^{-\frac{\varepsilon_P^{NS}+\varepsilon_{A_1}^{S}+\varepsilon_{A_2}^{NS}+\varepsilon_{A_3}^{NS}}{k_B*T}}}{P! * (A_1-1)! * A_2! * A_3! * (N_{NS}-P-(A_1-1)-A_2-A_3)!}$$

$$Z(P, A_1, A_2-1, A_3) = \frac{N_{NS}! * e^{-\frac{\varepsilon_P^{NS}+\varepsilon_{A_1}^{NS}+\varepsilon_{A_2}^{S}+\varepsilon_{A_3}^{NS}}{k_B*T}}}{P! * A_1! * (A_2-1)! * A_3! * (N_{NS}-P-A_1-(A_2-1)-A_3)!}$$

$$Z(P, A_1, A_2, A_3-1) = \frac{N_{NS}! * e^{-\frac{\varepsilon_P^{NS}+\varepsilon_{A_1}^{NS}+\varepsilon_{A_2}^{NS}+\varepsilon_{A_3}^{S}}{k_B*T}}}{P! * A_1! * A_2! * (A_3-1)! * (N_{NS}-P-A_1-A_2-(A_3-1))!}$$

$$Z(P, A_1-1, A_2-1, A_3) = \frac{N_{NS}! * e^{-\frac{\varepsilon_P^{NS}+\varepsilon_{A_1}^{S}+\varepsilon_{A_2}^{S}+\varepsilon_{A_3}^{NS}+\varepsilon_{A_1A_2}}{k_B*T}}}{P! * (A_1-1)! * (A_2-1)! * A_3! * (N_{NS}-P-(A_1-1)-(A_2-1)-A_3)!}$$

$$Z(P, A_1-1, A_2, A_3-1) = \frac{N_{NS}! * e^{-\frac{\varepsilon_P^{NS}+\varepsilon_{A_1}^{S}+\varepsilon_{A_2}^{NS}+\varepsilon_{A_3}^{S}+\varepsilon_{A_1A_3}}{k_B*T}}}{P! * (A_1-1)! * A_2! * (A_3-1)! * (N_{NS}-P-(A_1-1)-A_2-(A_3-1))!}$$

$$Z(P, A_1, A_2-1, A_3-1) = \frac{N_{NS}! * e^{-\frac{\varepsilon_P^{NS}+\varepsilon_{A_1}^{NS}+\varepsilon_{A_2}^{S}+\varepsilon_{A_3}^{S}+\varepsilon_{A_2A_3}}{k_B*T}}}{P! * A_1! * (A_2-1)! * (A_3-1)! * (N_{NS}-P-A_1-(A_2-1)-(A_3-1))!}$$

$$Z(P, A_1-1, A_2-1, A_3-1) = \frac{N_{NS}! * e^{-\frac{\varepsilon_P^{NS}+\varepsilon_{A_1}^{S}+\varepsilon_{A_2}^{S}+\varepsilon_{A_3}^{S}+\varepsilon_{A_1A_2}+\varepsilon_{A_1A_3}+\varepsilon_{A_2A_3}}{k_B*T}}}{P! * (A_1-1)! * (A_2-1)! * (A_3-1)! * (N_{NS}-P-(A_1-1)-(A_2-1)-(A_3-1))!}$$

$$Z(P-1, A_1, A_2, A_3) = \frac{N_{NS}! * e^{-\frac{\varepsilon_P^{S}+\varepsilon_{A_1}^{NS}+\varepsilon_{A_2}^{NS}+\varepsilon_{A_3}^{NS}}{k_B*T}}}{(P-1)! * A_1! * A_2! * A_3! * (N_{NS}-(P-1)-A_1-A_2-A_3)!}$$

$$Z(P-1, A_1-1, A_2, A_3) = \frac{N_{NS}! * e^{-\frac{\varepsilon_P^{S}+\varepsilon_{A_1}^{S}+\varepsilon_{A_2}^{NS}+\varepsilon_{A_3}^{NS}+\varepsilon_{PA_1}}{k_B*T}}}{(P-1)! * (A_1-1)! * A_2! * A_3! * (N_{NS}-(P-1)-(A_1-1)-A_2-A_3)!}$$

$$Z(P-1, A_1, A_2-1, A_3) = \frac{N_{NS}! * e^{-\frac{\varepsilon_P^{S}+\varepsilon_{A_1}^{NS}+\varepsilon_{A_2}^{S}+\varepsilon_{A_3}^{NS}+\varepsilon_{PA_2}}{k_B*T}}}{(P-1)! * A_1! * (A_2-1)! * A_3! * (N_{NS}-(P-1)-A_1-(A_2-1)-A_3)!}$$

$$Z(P-1, A_1, A_2, A_3-1) = \frac{N_{NS}! * e^{-\frac{\varepsilon_P^{S}+\varepsilon_{A_1}^{NS}+\varepsilon_{A_2}^{NS}+\varepsilon_{A_3}^{S}+\varepsilon_{PA_3}}{k_B*T}}}{(P-1)! * A_1! * A_2! * (A_3-1)! * (N_{NS}-(P-1)-A_1-A_2-(A_3-1))!}$$

$$Z(P-1, A_1-1, A_2-1, A_3) = \frac{N_{NS}! * e^{-\frac{\varepsilon_P^{S}+\varepsilon_{A_1}^{S}+\varepsilon_{A_2}^{S}+\varepsilon_{A_3}^{NS}+\varepsilon_{PA_1}+\varepsilon_{PA_2}+\varepsilon_{A_1A_2}}{k_B*T}}}{(P-1)! * (A_1-1)! * (A_2-1)! * A_3! * (N_{NS}-(P-1)-(A_1-1)-(A_2-1)-A_3)!}$$

$$Z(P-1, A_1-1, A_2, A_3-1) = \frac{N_{NS}! * e^{-\frac{\varepsilon_P^{S}+\varepsilon_{A_1}^{S}+\varepsilon_{A_2}^{NS}+\varepsilon_{A_3}^{S}+\varepsilon_{PA_1}+\varepsilon_{PA_3}+\varepsilon_{A_1A_3}}{k_B*T}}}{(P-1)! * (A_1-1)! * A_2! * (A_3-1)! * (N_{NS}-(P-1)-(A_1-1)-A_2-(A_3-1))!}$$

$$Z(P-1, A_1, A_2-1, A_3-1) = \frac{N_{NS}! * e^{-\frac{\varepsilon_P^{S}+\varepsilon_{A_1}^{NS}+\varepsilon_{A_2}^{S}+\varepsilon_{A_3}^{S}+\varepsilon_{PA_2}+\varepsilon_{PA_3}+\varepsilon_{A_2A_3}}{k_B*T}}}{(P-1)! * A_1! * (A_2-1)! * (A_3-1)! * (N_{NS}-(P-1)-A_1-(A_2-1)-(A_3-1))!}$$

$$Z(P-1, A_1-1, A_2-1, A_3-1) = \frac{N_{NS}! * e^{-\frac{\varepsilon_P^{S}+\varepsilon_{A_1}^{S}+\varepsilon_{A_2}^{S}+\varepsilon_{A_3}^{S}+\varepsilon_{PA_1}+\varepsilon_{PA_2}+\varepsilon_{PA_3}+\varepsilon_{A_1A_2}+\varepsilon_{A_1A_3}+\varepsilon_{A_2A_3}}{k_B*T}}}{(P-1)! * (A_1-1)! * (A_2-1)! * (A_3-1)! * (N_{NS}-(P-1)-(A_1-1)-(A_2-1)-(A_3-1))!}$$

Here, N_{NS} is the number of non-specific binding sites in the genome. The Boltzmann weights ε_i^S characterize specific binding of protein i to the promoter, while ε_i^{NS} describes non-specific binding to the genomic background. Transcription factor complexes with RNAP and other transcription factors are described by the weights ε_{PA_i} and $\varepsilon_{A_iA_j}$, respectively.

Using Eqs. S2.2 – S2.4, the probability of polymerase binding (Eq. S2.1) can be rewritten as (S2.5)

$$p_{bound} = \frac{\sum Z_{bound}}{Z_{tot}} = \frac{1}{1+\frac{\sum Z_{free}}{\sum Z_{bound}}} = \frac{1}{1+\frac{1}{F_{Reg}}*\frac{Z(P,A_1,A_2,A_3)}{Z(P-1,A_1,A_2,A_3)}} \approx \frac{1}{1+\frac{N_{NS}}{P*F_{Reg}}*e^{\frac{\Delta\varepsilon_P}{k_B*T}}}$$

In the last step, we calculated the ratio of $Z(P,A_1,A_2,A_3)$ and $Z(P-1,A_1,A_2,A_3)$, and additionally assumed that the number of polymerase and transcription factor molecules is much smaller than the total number of non-specific binding sites in the genome ($N_{NS} >> P$, $N_{NS} >> A_1$, $N_{NS} >> A_2$, $N_{NS} >> A_3$). This leads to the following approximation (S2.6)

$$\frac{Z(P,A_1,A_2,A_3)}{Z(P-1,A_1,A_2,A_3)} = \frac{N_{NS}-(P-1)-A_1-A_2-A_3}{P} * e^{\frac{\Delta\varepsilon_P}{k_B*T}} \approx \frac{N_{NS}}{P} * e^{\frac{\Delta\varepsilon_P}{k_B*T}}$$

Additionally, we used the notation $\Delta\varepsilon_P = \varepsilon_P^S - \varepsilon_P^{NS}$, and will use similar definitions to describe specific vs. non-specific binding of other proteins below. The regulation factor in Eq. S2.5 is given by (S2.7)

$$F_{Reg} = \frac{\sum Z_{bound}/Z(P-1,A_1,A_2,A_3)}{\sum Z_{free}/Z(P,A_1,A_2,A_3)}$$

Using Eq. S2.4, we obtain (S2.8)

$$F_{Reg} = \frac{1+c_1+c_2+c_3+c_4+c_5+c_6+c_7}{1+c_8+c_9+c_{10}+c_{11}+c_{12}+c_{13}+c_{14}}$$

where (S2.9)

$$c_1 = \frac{Z(P-1,A_1-1,A_2,A_3)}{Z(P-1,A_1,A_2,A_3)} = \frac{A_1}{N_{NS}} * e^{-\frac{\Delta\varepsilon_{A_1}}{k_B*T}} * e^{-\frac{\varepsilon_{PA_1}}{k_B*T}}$$

$$c_2 = \frac{Z(P-1,A_1,A_2-1,A_3)}{Z(P-1,A_1,A_2,A_3)} = \frac{A_2}{N_{NS}} * e^{-\frac{\Delta\varepsilon_{A_2}}{k_B*T}} * e^{-\frac{\varepsilon_{PA_2}}{k_B*T}}$$

$$c_3 = \frac{Z(P-1,A_1,A_2,A_3-1)}{Z(P-1,A_1,A_2,A_3)} = \frac{A_3}{N_{NS}} * e^{-\frac{\Delta\varepsilon_{A_3}}{k_B*T}} * e^{-\frac{\varepsilon_{PA_3}}{k_B*T}}$$

$$c_4 = \frac{Z(P-1,A_1-1,A_2-1,A_3)}{Z(P-1,A_1,A_2,A_3)} = \frac{A_1}{N_{NS}} * e^{-\frac{\Delta\varepsilon_{A_1}}{k_B*T}} * \frac{A_2}{N_{NS}} * e^{-\frac{\Delta\varepsilon_{A_1}}{k_B*T}} * e^{-\frac{\varepsilon_{PA_1}+\varepsilon_{PA_2}+\varepsilon_{A_1A_2}}{k_B*T}}$$

$$c_5 = \frac{Z(P-1, A_1-1, A_2, A_3-1)}{Z(P-1, A_1, A_2, A_3)} = \frac{A_1}{N_{NS}} * e^{-\frac{\Delta\varepsilon_{A_1}}{k_B*T}} * \frac{A_3}{N_{NS}} * e^{-\frac{\Delta\varepsilon_{A_3}}{k_B*T}} * e^{-\frac{\varepsilon_{PA_1}+\varepsilon_{PA_3}+\varepsilon_{A_1A_3}}{k_B*T}}$$

$$c_6 = \frac{Z(P-1, A_1, A_2-1, A_3-1)}{Z(P-1, A_1, A_2, A_3)} = \frac{A_2}{N_{NS}} * e^{-\frac{\Delta\varepsilon_{A_2}}{k_B*T}} * \frac{A_3}{N_{NS}} * e^{-\frac{\Delta\varepsilon_{A_3}}{k_B*T}} * e^{-\frac{\varepsilon_{PA_2}+\varepsilon_{PA_3}+\varepsilon_{A_2A_3}}{k_B*T}}$$

$$c_7 = \frac{Z(P-1, A_1-1, A_2-1, A_3-1)}{Z(P-1, A_1, A_2, A_3)} = \frac{A_1}{N_{NS}} * e^{-\frac{\Delta\varepsilon_{A_1}}{k_B*T}} * \frac{A_2}{N_{NS}} * e^{-\frac{\Delta\varepsilon_{A_2}}{k_B*T}} * \frac{A_3}{N_{NS}} * e^{-\frac{\Delta\varepsilon_{A_3}}{k_B*T}} * e^{-\frac{\varepsilon_{PA_1}+\varepsilon_{PA_2}+\varepsilon_{PA_3}+\varepsilon_{A_1A_2}+\varepsilon_{A_1A_3}+\varepsilon_{A_2A_3}}{k_B*T}}$$

$$c_8 = \frac{Z(P, A_1-1, A_2, A_3)}{Z(P, A_1, A_2, A_3)} = \frac{A_1}{N_{NS}} * e^{-\frac{\Delta\varepsilon_{A_1}}{k_B*T}}$$

$$c_9 = \frac{Z(P, A_1, A_2-1, A_3)}{Z(P, A_1, A_2, A_3)} = \frac{A_2}{N_{NS}} * e^{-\frac{\Delta\varepsilon_{A_2}}{k_B*T}}$$

$$c_{10} = \frac{Z(P, A_1, A_2, A_3-1)}{Z(P, A_1, A_2, A_3)} = \frac{A_3}{N_{NS}} * e^{-\frac{\Delta\varepsilon_{A_3}}{k_B*T}}$$

$$c_{11} = \frac{Z(P, A_1-1, A_2-1, A_3)}{Z(P, A_1, A_2, A_3)} = \frac{A_1}{N_{NS}} * e^{-\frac{\Delta\varepsilon_{A_1}}{k_B*T}} * \frac{A_2}{N_{NS}} * e^{-\frac{\Delta\varepsilon_{A_1}}{k_B*T}} * e^{-\frac{\varepsilon_{A_1A_2}}{k_B*T}}$$

$$c_{12} = \frac{Z(P, A_1-1, A_2, A_3-1)}{Z(P, A_1, A_2, A_3)} = \frac{A_1}{N_{NS}} * e^{-\frac{\Delta\varepsilon_{A_1}}{k_B*T}} * \frac{A_3}{N_{NS}} * e^{-\frac{\Delta\varepsilon_{A_3}}{k_B*T}} * e^{-\frac{\varepsilon_{A_1A_3}}{k_B*T}}$$

$$c_{13} = \frac{Z(P, A_1, A_2-1, A_3-1)}{Z(P, A_1, A_2, A_3)} = \frac{A_2}{N_{NS}} * e^{-\frac{\Delta\varepsilon_{A_2}}{k_B*T}} * \frac{A_3}{N_{NS}} * e^{-\frac{\Delta\varepsilon_{A_3}}{k_B*T}} * e^{-\frac{\varepsilon_{A_2A_3}}{k_B*T}}$$

$$c_{14} = \frac{Z(P, A_1-1, A_2-1, A_3-1)}{Z(P, A_1, A_2, A_3)} = \frac{A_1}{N_{NS}} * e^{-\frac{\Delta\varepsilon_{A_1}}{k_B*T}} * \frac{A_2}{N_{NS}} * e^{-\frac{\Delta\varepsilon_{A_2}}{k_B*T}} * \frac{A_3}{N_{NS}} * e^{-\frac{\Delta\varepsilon_{A_3}}{k_B*T}} * e^{-\frac{\varepsilon_{A_1A_2}+\varepsilon_{A_1A_3}+\varepsilon_{A_2A_3}}{k_B*T}}$$

By lumping constant terms together, we can rewrite Eqs. S2.5 and S2.8 as (S2.10)

$$F_{Reg} = \frac{1+\frac{[A_1]}{K_{A_1}}f_1+\frac{[A_2]}{K_{A_2}}f_2+\frac{[A_3]}{K_{A_3}}f_3+\frac{[A_1][A_2]}{K_{A_1}K_{A_2}}f_1f_2\omega_{12}+\frac{[A_1][A_3]}{K_{A_1}K_{A_3}}f_1f_3\omega_{13}+\frac{[A_2][A_3]}{K_{A_2}K_{A_3}}f_2f_3\omega_{23}+\frac{[A_1][A_2][A_3]}{K_{A_1}K_{A_2}K_{A_3}}f_1f_2f_3\omega_{12}\omega_{13}\omega_{23}}{1+\frac{[A_1]}{K_{A_1}}+\frac{[A_2]}{K_{A_2}}+\frac{[A_3]}{K_{A_3}}+\frac{[A_1][A_2]}{K_{A_1}K_{A_2}}\omega_{12}+\frac{[A_1][A_3]}{K_{A_1}K_{A_3}}\omega_{13}+\frac{[A_2][A_3]}{K_{A_2}K_{A_3}}\omega_{23}+\frac{[A_1][A_2][A_3]}{K_{A_1}K_{A_2}K_{A_3}}\omega_{12}\omega_{13}\omega_{23}}$$

$$p_{bound} = \frac{F_{Reg}}{F_{Reg}+K_P}$$

Here, the parameters K_{A_i} and K_P characterize specific vs. non-specific transcription factor or polymerase binding to DNA (S2.11).

$$K_{A_i} = N_{NS} * e^{\frac{\Delta\varepsilon_{A_2}}{k_B*T}}$$

$$K_P = \frac{N_{NS}}{P} * e^{\frac{\Delta\varepsilon_P}{k_B*T}}$$

Protein-protein complexes are described by ω_{ij} and f_l which determine interactions between transcription factors, and transcription factors and RNAP, respectively (S2.12).

$$\omega_{ij} = e^{-\frac{\varepsilon_{A_i A_j}}{k_B * T}}$$

$$f_i = e^{-\frac{\varepsilon_{P A_i}}{k_B * T}}$$

Luciferase expression was modeled using Eq. S2.10, assuming that steady state expression is proportional to the transcription initiation rate (see Section VI for details). The transcription factor concentrations $[A_i]$ represent the phospho-SMAD and phospho-STAT input into the promoter model, and were modeled as described in the following.

1. Hentze MW, Muckenthaler MU, Galy B, Camaschella C (2010) Two to tango: regulation of Mammalian iron metabolism. Cell 142: 24-38.
2. Bintu L, Buchler NE, Garcia HG, Gerland U, Hwa T, et al. (2005) Transcriptional regulation by the numbers: applications. Current opinion in genetics & development 15: 125-135.
3. Bintu L, Buchler NE, Garcia HG, Gerland U, Hwa T, et al. (2005) Transcriptional regulation by the numbers: models. Current opinion in genetics & development 15: 116-124.
4. Sherman MS, Cohen BA (2012) Thermodynamic state ensemble models of cis-regulation. PLoS computational biology 8: e1002407.
