# Supplementary material for: A Multi-Scale Model of Hepcidin Promoter Regulation Reveals Factors Controlling Systemic Iron Homeostasis
Source: PLoS Comput Biol. 2014 Jan 2;10(1):e1003421. doi: 10.1371/journal.pcbi.1003421 (PMC3879105; doi:10.1371/journal.pcbi.1003421)
Supplement: Text S3 — Modeling the dose-response behavior of signaling pathways. (PDF) [file pcbi.1003421.s014.pdf]

Supplemental Text S3 – Modeling the dose-response behavior of signaling pathways

Model without signaling crosstalk: The dose-response of intracellular signaling pathways is typically sigmoidal in shape, and described by the Hill equation ($y = y_{basal} + y_{max} * S^n/(S^n + EC_{50}{}^n)$) [1,2]. The Hill equation expresses signaling (y) as a sigmoidal function of the stimulus (S), and takes into account basal signaling (y_{basal}), maximal pathway activation (y_{max}), the half-maximal-stimulus (EC50) and the Hill coefficient (n) as a measure of steepness of the dose-response curve. In our initial model, we neglected inhibitory crosstalk between BMP and IL6 pathways, and described the steady state of each signaling cascade using a Hill equation. The concentrations of phospho-STAT (pST) and phospho-SMAD (pSM) are thus described by (S3.1)

$$[pST] = y_{max,1} * f_{Hill,IL6}$$

$$[pSM] = y_{basal,2} + y_{max,2} * f_{Hill,BMP}$$

where $f_{Hill,IL6} = \frac{[IL6]^{n_1}}{[IL6]^{n_1} + EC_{50,1}{}^{n_1}}$ and $f_{Hill,BMP} = \frac{[BMP]^{n_2}}{[BMP]^{n_2} + EC_{50,2}{}^{n_2}}$

The output concentrations of these Hill equations (pSM and pST) were in turn used as an input for the thermodynamic promoter model (see Supplemental Protocol S3).

Model including signaling crosstalk: During our experimental analyses, we observed that the IL6 and BMP mutually inhibit each other at the level of transcription factor phosphorylation, although to a minor extent (Fig. 1D and Supplemental Fig. S2). We therefore extended our initial signaling model, and took inhibitory crosstalk into account.

The main objective of the signaling crosstalk model was to represent the existing data (Fig. 1D and Supplemental Fig. S2), and to extrapolate the concentrations of phosphorylated transcription factors for conditions where experimental measurements were not available: STAT phosphorylation was only assessed for increasing doses of IL6 in the presence or absence of saturating amounts of BMP (800 ng/ml). Likewise, SMAD phosphorylation was monitored for increasing doses of BMP, alone or in combination with 25 ng/ml IL6 (Figs. 2C and S4). Since luciferase expression was additionally assessed for intermediate IL6 and BMP doses, less input than output conditions are known in the promoter model. To overcome this problem, we estimated transcription factor activity using a model-based extrapolation strategy.

A model describing signaling crosstalk should be able to simultaneously describe dose-response curves of pSMAD and pSTAT in the presence or absence of the non-canonical inhibitory stimulus (IL6 and BMP, respectively). To investigate the mode of crosstalk regulation, we fitted the Hill equation to the dose-response data (Fig. 1D). Different

scenarios of signaling crosstalk were analyzed: the non-canonical stimulus (i.e., BMP for STAT and IL6 for SMAD) was assumed to affect dose-response of transcription factor activation at the level of one or more parameters of the Hill equation (y_{basal}, y_{max}, EC50 and n). The fits in Fig. 1D reveal that crosstalk can be described quantitatively if it is assumed that non-canonical stimulation modulates that maximal activation level in the Hill equation (y_{max}). The assumption of selective crosstalk modulation at the level of y_{max} was used to derive a simple mathematical model for signaling crosstalk as described in the following.

In a simple crosstalk formulation the concentration of phosphorylated transcription factors can be written as (S3.2)

$$[pST] = \frac{y_{max,1}}{1 + k_{C,1} * [pSM]} * f_{Hill,IL6}$$

$$[pSM] = y_{basal,2} + \frac{y_{max,2} * f_{Hill,BMP}}{1 + k_{C,2} * [pST]}$$

where $f_{Hill,IL6} = \frac{[IL6]^{n_1}}{[IL6]^{n_1} + {EC_{50,1}}^{n_1}}$ and $f_{Hill,BMP} = \frac{[BMP]^{n_2}}{[BMP]^{n_2} + {EC_{50,2}}^{n_2}}$

For each transcription factor, the maximal activation level is reduced by the presence of the opposite factor (with crosstalk strength constants $k_{C,1}$ and $k_{C,2}$). We have taken into account that basal STAT phosphorylation is negligible in our experimental setup ($y_{basal,1} = 0$). We can solve for pSTAT by plugging in the pSMAD concentration, and obtain (S3.3)

$$[pST] = \frac{1}{2} * \frac{1}{k_{C,2}} * \left(\frac{k_{C,2} * y_{max,1} * f_{Hill,IL6} - \left(1 + k_{C,1} * \left(y_{basal,2} + y_{max,2} * f_{Hill,BMP}\right)\right)}{1 + k_{C,1} * y_{basal,2}} + \sqrt{\left(\frac{k_{C,2} * y_{max,1} * f_{Hill,IL6} - \left(1 + k_{C,1} * \left(y_{basal,2} + y_{max,2} * f_{Hill,BMP}\right)\right)}{1 + k_{C,1} * y_{basal,2}} \right)^2 + 4 * k_{C,2} * \frac{y_{max,1} * f_{Hill,IL6}}{1 + k_{C,1} * y_{basal,2}}} \right)$$

As expected, this expression simplifies to the normal Hill equation ($[pST] = y_{max,1} * f_{Hill,IL6}$) in the absence of SMAD signaling (i.e., if $y_{basal,2} = 0$ and $y_{max,2} = 0$).

This minimal crosstalk model was sufficient to accurately and simultaneously describe the phospho-STAT and phospho-SMAD dose-response curves (Figure 2C shows the fit of Eqs. S3.2 and S3.3 to the Western Blot data). The crosstalk model could extrapolate transcription factor phosphorylation for intermediate levels of BMP and IL6 that were not measured experimentally. Thus, the signaling crosstalk provided a complete input map for the promoter module.

1. Heinrich R, Neel BG, Rapoport TA (2002) Mathematical models of protein kinase signal transduction. Molecular cell 9: 957-970.
2. Ferrell JE, Jr. (1996) Tripping the switch fantastic: how a protein kinase cascade can convert graded inputs into switch-like outputs. Trends in biochemical sciences 21: 460-466.
