# Supplementary material for: A Multi-Scale Model of Hepcidin Promoter Regulation Reveals Factors Controlling Systemic Iron Homeostasis
Source: PLoS Comput Biol. 2014 Jan 2;10(1):e1003421. doi: 10.1371/journal.pcbi.1003421 (PMC3879105; doi:10.1371/journal.pcbi.1003421)
Supplement: Text S4 — Detailed model of systemic iron homeostasis. (PDF) [file pcbi.1003421.s015.pdf]

Supplemental Text S4 – Detailed model of systemic iron homeostasis

We sought to investigate how the experimentally verified model of signal integration at the hepcidin promoter affects systemic iron homeostasis. We therefore modified the homeostasis model (Eq. S1.1) to (S4.1)

$$\frac{d[Fe_b]}{dt} = \frac{k_{influx}}{1+k_{FB}\cdot[hepcidin]}\cdot[Fe_i] - k_{efflux}\cdot[Fe_b]$$

$$\frac{d[hepcidin]}{dt} = k_{induced} * p_{bound} - k_{deg}\cdot[hepcidin]$$

Here, p_{bound} equals the probability of RNAP being bound to the transcription start site according to the best-fit promoter and signaling crosstalk model (Eqs. S3.2, S3.3 and S2.13).

The best-fit promoter and signaling crosstalk model describes p_{bound} as a function of BMP and IL6 concentrations. In the animal, the activity of the BMP signaling pathway is controlled by the iron blood levels. For simplicity, we assumed that the effective BMP concentration in the body is proportional to the iron blood level. Thus, the BMP concentration entering p_{bound} was replaced by the species $[Fe_b]$ in the detailed model of systemic iron homeostasis. For the simulations in Fig. 4E, we chose the following kinetic parameters (k_{influx} = 1; k_{FB} = 100; k_{efflux} = 1; $k_{induced}$ = 1; k_{deg} = 1). Strong feedback (high k_{FB}) was assumed to ensure that the degree of iron homeostasis (determined by the fold-change in $[Fe_b]$ brought about by a certain fold-change in $[Fe_i]$) is solely determined by the steepness of the hepcidin promoter, and not by the other model parameters (Supplemental Text S1). The IL6 concentration was set to zero in most simulations, and assumed to be saturating in the dashed blue line in Fig. 4E.

The detailed model of systemic iron homeostasis describes how promoter mutations and IL6 co-stimulation affect the performance of the iron homeostasis loop. Fig. 4E shows corresponding simulations for one particular set of kinetic parameters. It should be noted that similar conclusions concerning the modulation of iron homeostasis continue to hold for other parameter values of k_{influx}, k_{FB}, k_{efflux}, $k_{induced}$ and k_{deg}. This is due to the fact that these parameter values affect the absolute iron blood levels in the model, but not the qualitative features of homeostasis (Supplemental Text S1).
